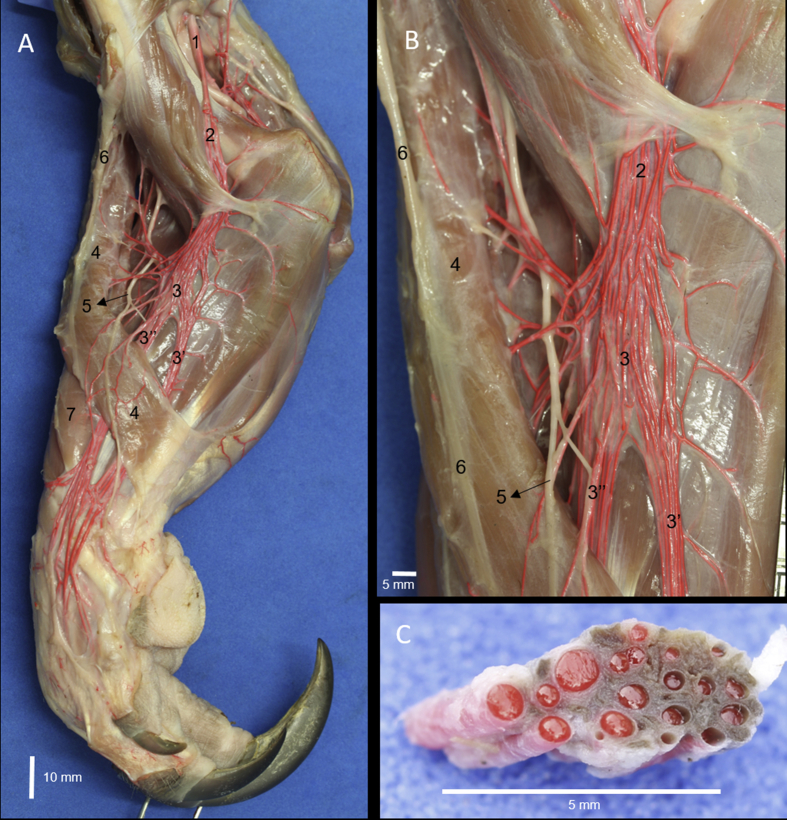# Gross anatomical adaptations of the craniolateral forearm muscles in *Tamandua mexicana* (Xenarthra: Myrmecophagidae): development of accessory muscles and *rete mirabile* for its arterial supply

**DOI:** 10.1016/j.heliyon.2019.e02179

**Published:** 2019-08-19

**Authors:** Paula Valentina Polania-Guzmán, Juan Fernando Vélez-García

**Affiliations:** Grupo de investigación en Medicina y Cirugía de Pequeños Animales, Programa de Medicina Veterinaria y Zootecnia Departamento de Sanidad Animal, Facultad de Medicina Veterinaria y Zootecnia, Universidad del Tolima, Ibagué, Colombia

**Keywords:** Brachioradialis muscle, Forelimb, Myology, Vermilingua, Zoology

## Abstract

The northern tamandua (*Tamandua mexicana*) is a xenarthran mammal with a distribution from Mexico to Peru. This species arrives to wildlife care centres due to illegal trafficking and attacks by domestic dogs, both of which are situations where the northern tamandua's thoracic limbs (forelimbs) can be affected. As such, it is necessary to have anatomical studies that allow us to perform better medical and surgical procedures. Among these, studies about the musculoskeletal system also aid in the muscular reconstructions of extinct species. The aim of this study was to characterize the craniolateral muscles of the forearm in *Tamandua mexicana* and compare them with other Xenarthrans to determine their gross adaptations. Six dead specimens were used, and none were sacrificed for the purpose of this investigation. In five specimens, arterial repletion was done. Four were fixed with 10% formaldehyde and 5% glycerin, and two were dissected in fresh. All were dissected in the Veterinary Anatomy Laboratory of the Universidad del Tolima. The weights of the muscles from seven forearms were taken and divided in three functional groups for comparison with non-parametric statistics. Two muscular groups were found: one superficial formed by the brachioradialis, brachioradialis accesorius, extensor carpi radialis, extensor digitorum communis, extensor digitorum lateralis and extensor carpi ulnaris; and one deep muscular group formed by the supinator, extensor digiti III et IV, abductor digiti I longus, and extensor digiti I et II. They were supplied by different branches of the cranial interosseous, transverse cubital and superficial brachial arteries, which had the shape of *rete mirabile*; and all muscles were innervated by the deep branch of the radial nerve. The presence of the brachioradialis accesorius muscle in this species allows its hand to remain in semi-supination when it is mobilized in a quadrupedal manner. It must also support elbow flexion together with the action of the brachioradialis and the extensor carpi radialis muscles. All the antebrachial digital muscles sent tendons for the digit III making it the most functional for different grip activities such as climbing trees and searching for its food, however, the most strength was directed to supination and carpal extension, and therefore also to the flexion of the elbow.

## 1 Introduction

The northern tamandua (*Tamandua mexicana*) belongs to the superorder Xenarthra and the family Myrmecophagidae, as it has characteristics that differentiate it from other mammals such as an extra joint in its lumbar vertebrae and its lack of teeth (Munao, 2001; Gaudin and McDonald, 2008; Hautier et al., 2018). It is distributed geographically from southern Mexico, reaching northwestern Colombia, northwestern Venezuela, western Ecuador and northwestern Peru (Tirira, 2007; Gardner, 2008; Superina et al., 2010; Nuñez-Perez et al., 2011; Brown, 2011). In Colombia, it has been registered from 0-1200 meters above sea level (Díaz et al., 1986), however, individuals have been reported up to 3000 meters above sea level (Calle and Arango, 2003) and can be found in the Andean, Pacific and Caribbean regions of Colombia (Díaz et al., 1986; Calle and Arango, 2003; Ramírez and Noguera, 2010). *Tamandua mexicana* is a species of solitary and territorial habits (Navarrete and Ortega, 2011), which marks its territory with anal odoriferous glands (Montgomery, 1985). They reproduce once a year at any time and their gestation period lasts between 130 and 150 days (Silveira, 1969), and the brood remains with its mother until it reaches one year of age (Nowak and Walker, 1999).

The species has an elongate toothless head, with a long tongue which is highly sticky due to saliva produced by the large mandibular gland (Reiss, 1997; Cuarón, 2014). The tongue has a sternoglossus muscle, used for protraction and full retraction (Reiss, 1997; Silva et al., 2016), and therefore is adapted for its diet based on the consumption of ants and termites (Sandoval-Gómez et al., 2012), although they have also been observed feeding upon fruit in a palm (*Attalea butyracea*) as a supplement to their insect diet (Brown, 2011). The hyoid apparatus is robust, and together with the larynx are in the caudal part of the neck, just cranial to sternal manubrium (Reiss, 1997), which prevents irregular movements during food intake (Silva et al., 2016).

The species has terrestrial and arboreal habits, and therefore, it presents anatomical characteristics in their locomotor limbs and tail (prehensile) that allow them to carry out activities such as feeding and moving in both environments (Montgomery, 1985; Navarrete and Ortega, 2011), even its ability to swim has been documented (Esser et al., 2010). The thoracic limbs (forelimbs) have four visible digits with great claws developing mainly on the digit third (Cuarón, 2014; Humanez-López and Chacón Pacheco, 2014), although in *Tamandua tetradactyla* it has been documented that the hand has five digits, however the digit fifth is not visible (Machado-Cruvinel et al., 2019). The pelvic limbs (Hindlimbs) have five digits of similar shape and adapted to plantigrade locomotion (Humanez-López and Chacón-Pacheco, 2014).

For locomotion, the bones and muscles have specific characteristics in their thoracic limb which differentiate the *Tamandua* genus widely from other mammal species, such as the scapula with two spines, a longer acromion, and a postscapular fossa; the humerus having a highly developed deltoid tuberosity and a delto-epicondylar ligament (Taylor, 1978); the bones for the digit III having special features such as a robust metacarpal bone, the proximal phalanx being extremely short and the distal phalanx being longer than other digits; the teres major muscle having wide attachments and the medial head of the triceps brachii muscle being fused with the flexor digitorum profundus muscle (Taylor, 1978). These characteristics are also important for their daily activities since these allow it to break loose hard materials to feed, defend themselves (Nowak and Walker, 1999), and in the male, it permits it to manipulate the position of the female during mating (Matlaga, 2006).

In some xenarthrans, such as sloths and anteaters (*Cyclopes* and *Tamandua*), the development of the arterial plexus in their limbs has been documented, being named rete mirabile (*Rete mirabile*) (Wislocki and Straus, 1932; Aguilar and Superina, 2015), but there are no detailed descriptions of the distribution of these arteries. However, the rete mirabile contributes to thermoregulation since it lows the temperature of the anatomical structure that it supplies and the adjacent structures (Scholander, 1955; Castellini, 2009), thus it is important to know the distribution of these plexuses in order to recognize their role in this part of the body.

The northern tamandua is on the IUCN Red List (International Union for Conservation of Nature and Natural Resources) as a species of the least concern (LC) category due to its widespread distribution and the wide population range of this species (Ortega-Reyes et al., 2014). However, it is constantly threatened by different factors, such as being hit by road vehicles, forest fires, changes in its habitat, illegal trafficking, attacks by domestic dogs in rural and peri-urban areas (Ortega-Reyes et al., 2014; Rojano, 2014), and, to our knowledge, by electric shocks. All of these situations that can generate injuries in their thoracic limbs, and therefore, it is common to find them in wildlife care centres. These situations make it necessary to have a specific knowledge of the gross anatomy of the northern tamandua, which will be useful for wildlife veterinarians to carry out successful clinical and surgical procedures that contribute to their medical recuperation. Studies about the musculoskeletal system also will aid in the muscular reconstructions of extinct xenarthran species (Vizcaíno and Bargo, 2003; McDonald et al., 2008; Toledo et al., 2013; Vizcaíno et al., 2017; Amson and Nyakatura, 2018), therefore, the main objective of this study is to characterize the craniolateral muscles of the forearm in *Tamandua mexicana,* reporting in detail anatomical characteristics such as shape, origin, insertion, innervation and arterial supply, which will aid in comparisons with other Xenarthrans species to determine their gross anatomical adaptations to satisfy with different actions in the elbow and hand, and will be useful for some surgical approaches in the humerus, radius and ulna.

Although there are anatomical reports about these muscles in species of the suborder Vermilingua such as *Tamandua tetradactyla* (Cuvier and Laurillard, 1850; Windle and Parsons, 1899), *Tamandua* sp*.* (Taylor, 1978), *Myrmecophaga tridactyla* (Macalister, 1875a) and *Cyclopes didactylus* (Galton, 1869a; Humphry, 1869), these studies have the following disadvantages: they are not complete descriptions of the muscles, the terminology used is obsolete, there is a lack of innervation and arterial supply in the descriptions, and none of these reports specifically address *Tamandua mexicana.* Therefore, with this study we also intend to overcome these deficiencies in the knowledge of the craniolateral muscles of the forearm in this species and it will be compared with other xenarthran species, especially with others vermilinguas. We also compare with domestic carnivores as they have five digits and their anatomy is the most studied from the veterinary point of view since they are the base species to perform clinical and surgical approaches in wild animals.

## 2 Materials and methods

### 2.1 Specimens

This study was approved by the bioethics committee of the Universidad del Tolima (2.3–059). The thoracic limbs from six cadavers of *T. mexicana,* among them two females and four males, without history of musculoskeletal disorders were used. Four specimens died from natural causes between 2015 and 2018 in the wildlife care centres of CORTOLIMA (Corporación Autónoma Regional del Tolima, environmental authority of Tolima - Colombia). These specimens were donated to the Laboratory of Veterinary Anatomy of the Universidad del Tolima. Both separated thoracic limbs of two males were borrowed for this study from the Laboratory of Veterinary Anatomy of the Universidad Nacional de Colombia (Bogotá). The bones of the thoracic limbs from three specimens (two males and one female) had physes in the radiographs, therefore these specimens were considered juveniles.

### 2.2 Conservation and fixation

After necropsy, half of the body of a female (head, neck, thorax and thoracic limbs) and both thoracic limbs of two males were separated and frozen. The complete corpse of a male specimen was frozen. The other two specimens (one complete female and one necropsied male) were fixed using subcutaneous and intramuscular routes with a solution of 10% formaldehyde and 5% glycerin. Subsequently they were conserved by submersion in a 10% formaldehyde solution for at least one week.

### 2.3 Arterial repletion

After complete defrosting, arterial repletion was performed with natural latex tinctured with red vinyl via axillary arteries in the separated thoracic limbs; in the female with only half a body, the repletion was done via the thoracic aorta; in the complete corpse of a male it was done via the femoral artery; and in the other corpse (female) the repletion was done two weeks after of its fixation. The arterial repletion was not done only in one necropsied corpse (male). Posterior to the repletion, the specimens were frozen again for at least two weeks after which they were fixed posteriorly with the prior mentioned solution.

### 2.4 Dissection and documentation

Gross dissections were made in the Laboratory of Anatomy Veterinary of the Universidad del Tolima, where both thoracic limbs were dissected from superficial to deep, emphasizing the craniolateral part of the forearm (*Regio antebrachii*), in which the shape, origin, insertion, innervation and arterial supply of the craniolateral forearm muscles were described. Photographs were taken during the process of dissection with a camera CANON T5i (Canon Inc., Tokyo, Japan) associated with a macro lens of 60 mm and with a Canon power shot elph 150 Is, 18 mp. The anatomical characteristics were described according to the terminology of the *Nomina Anatomica Veterinaria* (International Committee on Veterinary Gross Anatomical Nomenclature, 2017).

### 2.5 Weighting of muscles

The craniolateral forearm muscles fixed in formalin of seven limbs were retired and weighed to compare three functional groups: Carpal extensors, digital extensors and supinators. The weight of each muscle was taken in an analytical scale Explorer Pro (Ohaus®) EP213 (Max 210g, d = 1mg). The proportions of each muscle in the craniolateral forearm musculature were calculated from the averages of the total weight (Table 1).

**Table 1:** Weights (grams) and proportions of the craniolateral forearm muscles in *Tamandua mexicana*.

Muscle	Rigth forearm	Left forearm	Rigth forearm	Left forearm	Rigth forearm	Left forearm	Rigth Forearm	Average	Proportion (%)
	S1 (M)	S2 (jM)	S2 (jM)	S3 (F)	S3 (F)	S6 (F)	S6 (F)		
Brachioradialis	5.269	4.319	3.843	2.792	2.769	5.194	5.508	4.242	10.050
Brachioradialis accessorius	9.791	10.176	9.142	7.548	7.324	10.124	10.504	9.230	21.860
Extensor carpi radialis	14.786	12.676	12.478	11.569	11.592	17.377	17.509	13.998	33.160
Extensor digitorum communis	5.324	6.742	6.311	4.832	4.789	7.749	7.006	6.108	14.470
Extensor digitorum lateralis	0.909	0.357	0.516	0.649	0.445	0.880	0.768	0.603	1.430
Extensor carpi ulnaris	5.929	5.541	5.422	4.992	4.748	7.469	7.413	5,931	14.050
Extensor digiti III et IV	0.367	0.250	0.352	0.203	0.243	0.355	0.566	0.334	0.790
Extensor digiti I et II	1.533	1.039	0.891	0.553	0.769	1.461	1.614	1.123	2.660
Abductor digiti I longus	3.984	3.365	2.521	2.707	2.775	4.310	3.607	3.324	7.870
Supinator	2.586	2.660	1.899	2.001	1.876	3.085	2.920	2.432	5.760
Total	14.399	47.125	43.375	37.846	37.330	58.004	57.415	42.213	100.000

S: specimen; M: male; jM: juvenile male; F: female.

### 2.6 Statistical analysis

The weights were tabulated and analyzed using descriptive statistics. The mean, standard deviation, median, range, minimum value and maximum value were obtained (Table 2). The statistical differences among the weights of the functional groups were evaluated using the Kruskal-Wallis test at an α of 0.05 with the software XLSTAT (Addinsoft, Barcelona) (Tables 3 and 4).

**Table 2:** Descriptive statistics of each functional muscular group in the craniolateral forearm muscles.

Stats	Digital extensors	Carpal extensors	Supinators
Number of comparisons	28	21	21
Minimum	0.055	2.521	1.876
Maximum	7.469	17.509	10.504
Rank	7.414	14.988	8.628
1° Quartile	0.365	3.984	2.792
Median	0.825	5.541	5.194
3° Quartile	2.408	11.592	7.769
Mean	2.025	7.751	5.537
Standard deviation (n)	2.417	4.8	3.023

**Table 3:** P-value.

	Supinators	Carpal extensors	Digital extensors
Supinators	1	0.326	**0**
Carpal extensors	0.326	1	<0.0001
Digital extensors	0	<0.0001	1

Bonferroni-corrected p-value: *0.0167*.

**Table 4:** Dunn's multiple comparison test with multiple comparisons/bilateral test.

Sample	Frequency	Sum of ranges	Mean	Groups	
Digital extensors	28	565.5	20.196	A	
Supinators	21	895	42.619		B
Carpal extensors	21	1024.5	48.786		B

### 2.7 Limitations of the study

Only six specimens were studied as they were the only ones donated for the present study between 2015 and 2018. Among these, the muscles of two fresh males and one limb of a fixed male specimen were not weighed because they were poorly preserved, and the full weight of all specimens was not provided by the donors. However, the others basic measurements were taken in three fixed specimens with a measuring tape (Table 5). Only three measurements were provided by the donor in one female specimen (S6) (Table 5).

**Table 5:** Basic measurements (cm) in four specimens of *T. mexicana*.

Measurements (cm)	Specimen 1 (M)	Specimen 2 (jM)	Specimen 3 (F)	Specimen 6 (F)
Head body length	62	54	58	∗
Body length	47	43	46	∗
Tail length	61	57	50	∗
Total length	123	111	108	125
Forelimb length	32	31	30	30
Hindlimb length	34	32	28	31

∗ Missing measurements, M: male, jM: juvenile male, F: Female.

## 3 Results

**Fig. 1:**
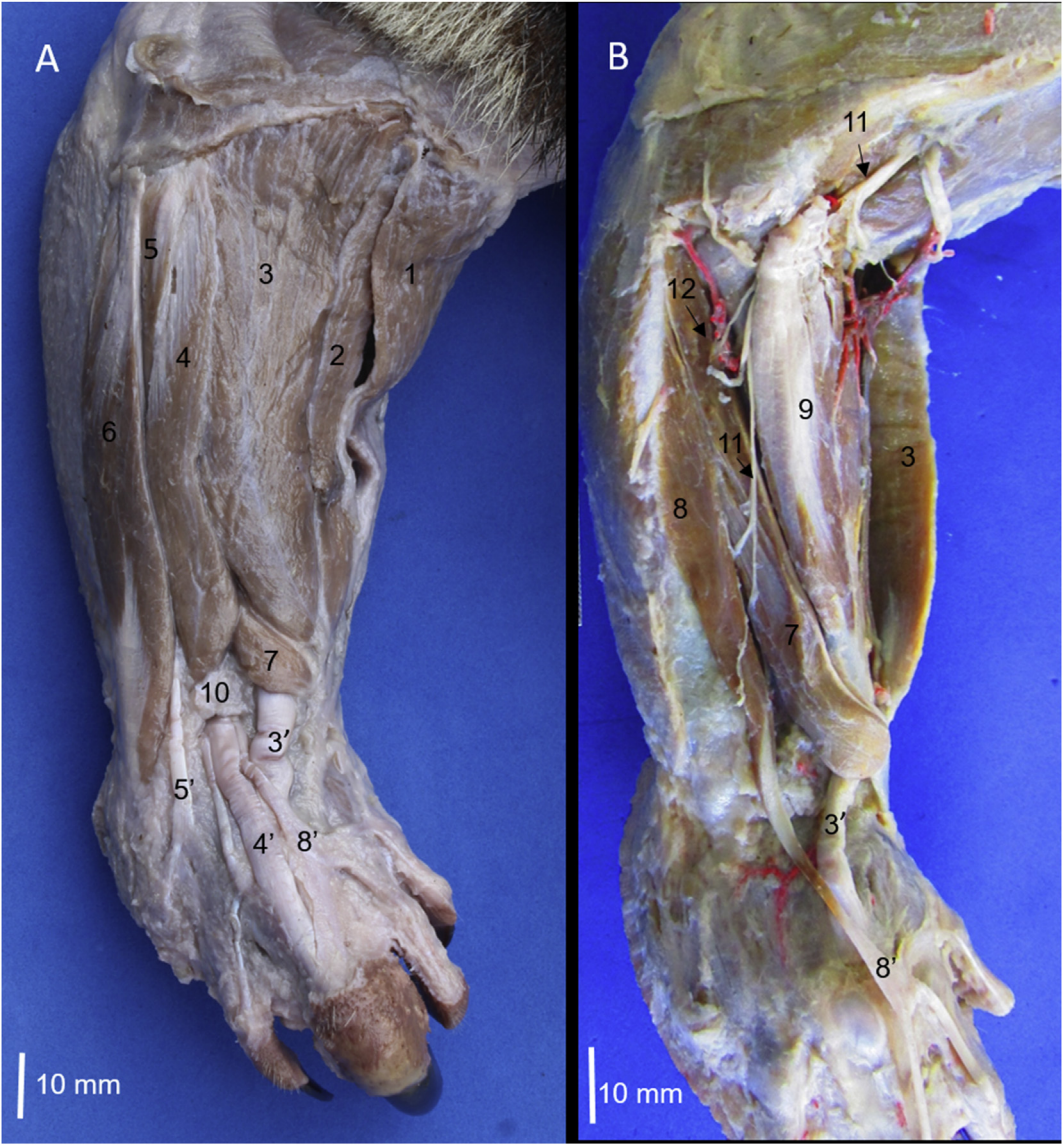
Craniolateral muscles of the right forearm in *Tamandua mexicana*. A) superficial group, B) deep group. 1) m. brachioradialis accesorium, 2) m. brachioradialis, 3) m. extensor carpi radialis, 4) m. extensor digitorum communis, 5) m. extensor digitorum lateralis, 6) m. extensor carpi ulnaris, 7) m. abductor longus I digiti, 8) m. extensor digiti I et II, 9) m. supinator, 10) extensor retinaculum, 3′-5′,8′) tendons of the respective muscle, 10) extensor retinaculum, 11) deep branch of the radial nerve, 12) cranial interosseus artery (arterial plexus).

**Fig. 2:**
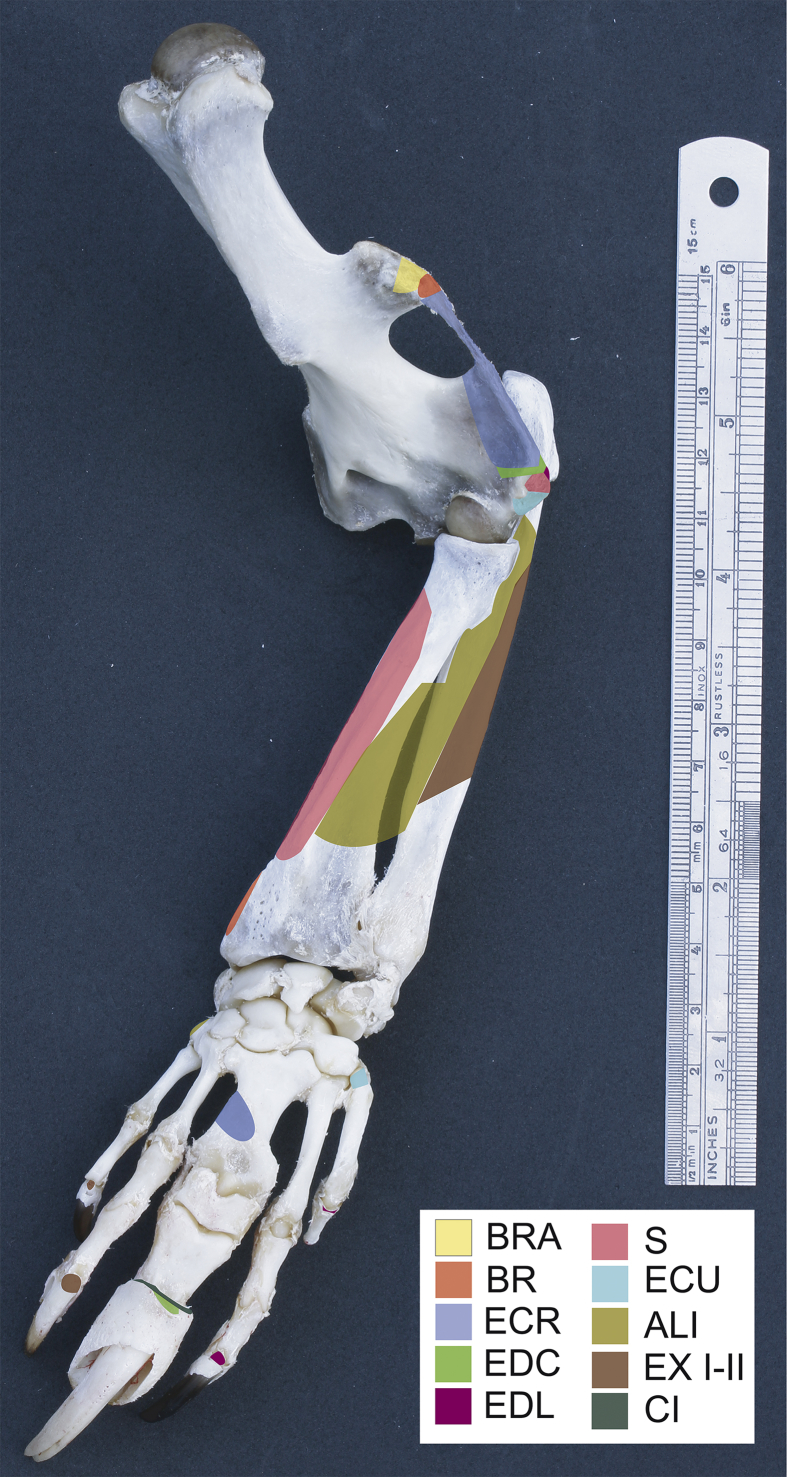
Origins and insertions of the craniolateral forearm muscles in the bones of the thoracic limb (left). BRA) brachioradialis accesorius, BR) brachioradialis, ECR) extensor carpi radialis, EDC) extensor digitorum communis, EDL) extensor digitorum lateralis, S) supinator, ECU) extensor carpi ulnaris, ALI) abductor digiti I longus, (EX I-II) extensor digiti I et II, CI) Common insertion.

The craniolateral muscles of the forearm of *Tamandua mexicana* are divided into superficial and deep groups. The superficial group was formed by the brachioradialis, brachioradialis accessorius, extensor carpi radialis, extensor digitorum communis, extensor digitorum lateralis and extensor carpi ulnaris muscles (Figs. 1A, 2). Whereas the deep group is formed by the supinator, extensor digiti I et II, extensor digiti III et IV, and abductor digiti I longus muscles (Figs. 1 and 2).

### 3.1 Descriptive myology

**Fig. 3:**
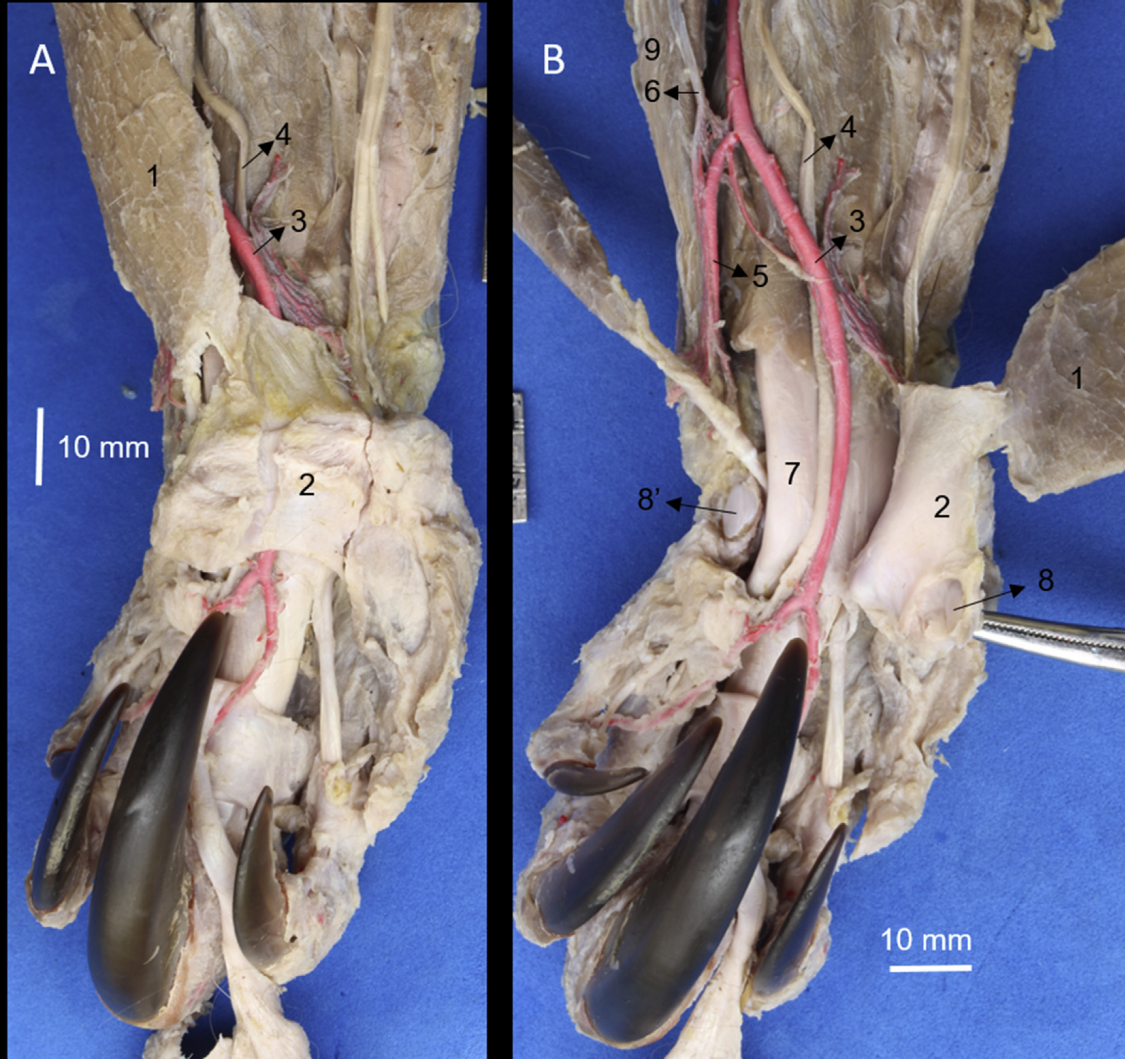
A) Caudal view of the extreme distal of the right forearm and palmar view. B) the m. brachioradialis accesorius has been retracted laterally. 1) m. brachioradialis accesorius, 2) flexor retinaculum, 3) median artery, 4) median nerve, 5) radial artery, 6) medial branch of the superficial cranial antebrachial artery, 7) tendon of the m. flexor digitorum profundus, 8) sessamoid bone of the flexor retinaculum, 8′) articular surface of the radial carpal bone for 8, 9) m. pronator teres.

**Fig. 4:**
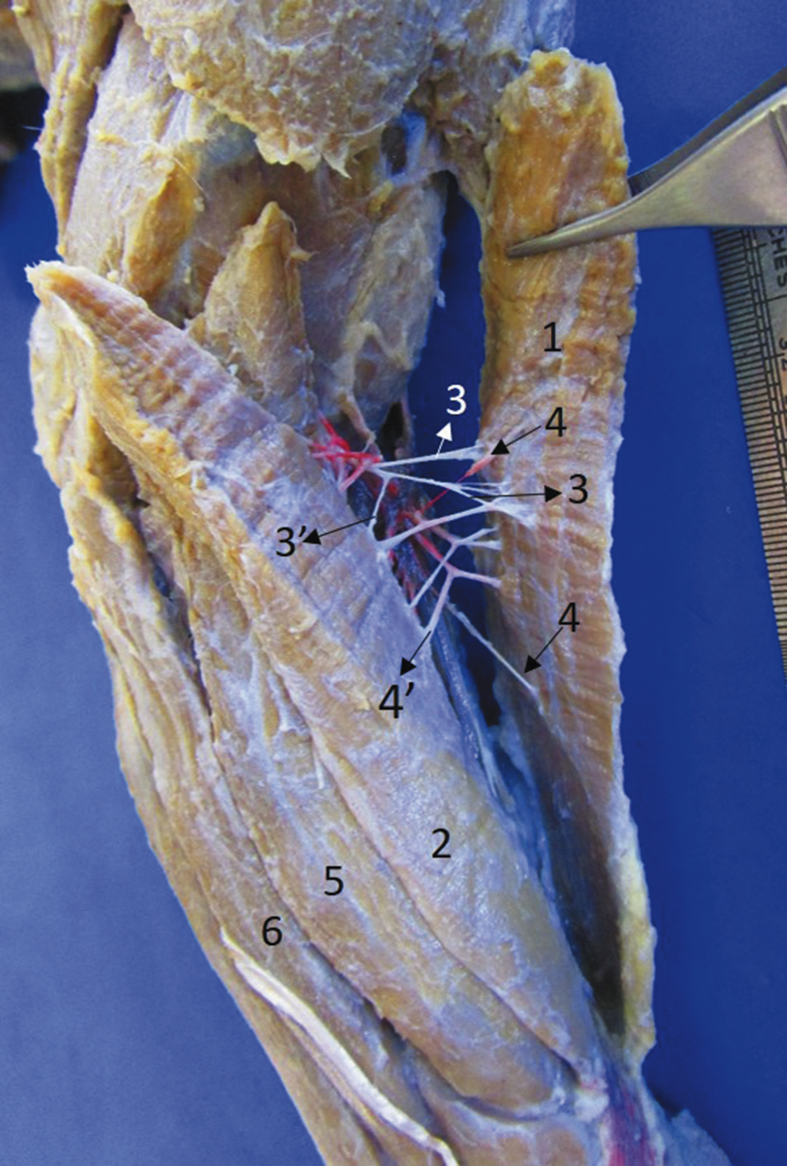
1) Brachioradialis accesorius, 2) brachioradialis, 3) branches of the radial nerve to 1, 3′) branch of the radial nerve to 2, 4) branches of the superficial brachial artery to 1, 4′) branches of the superficial brachial artery to 3, 5) extensor carpi radialis, 6) extensor digitorum communis.

**Fig. 5:**
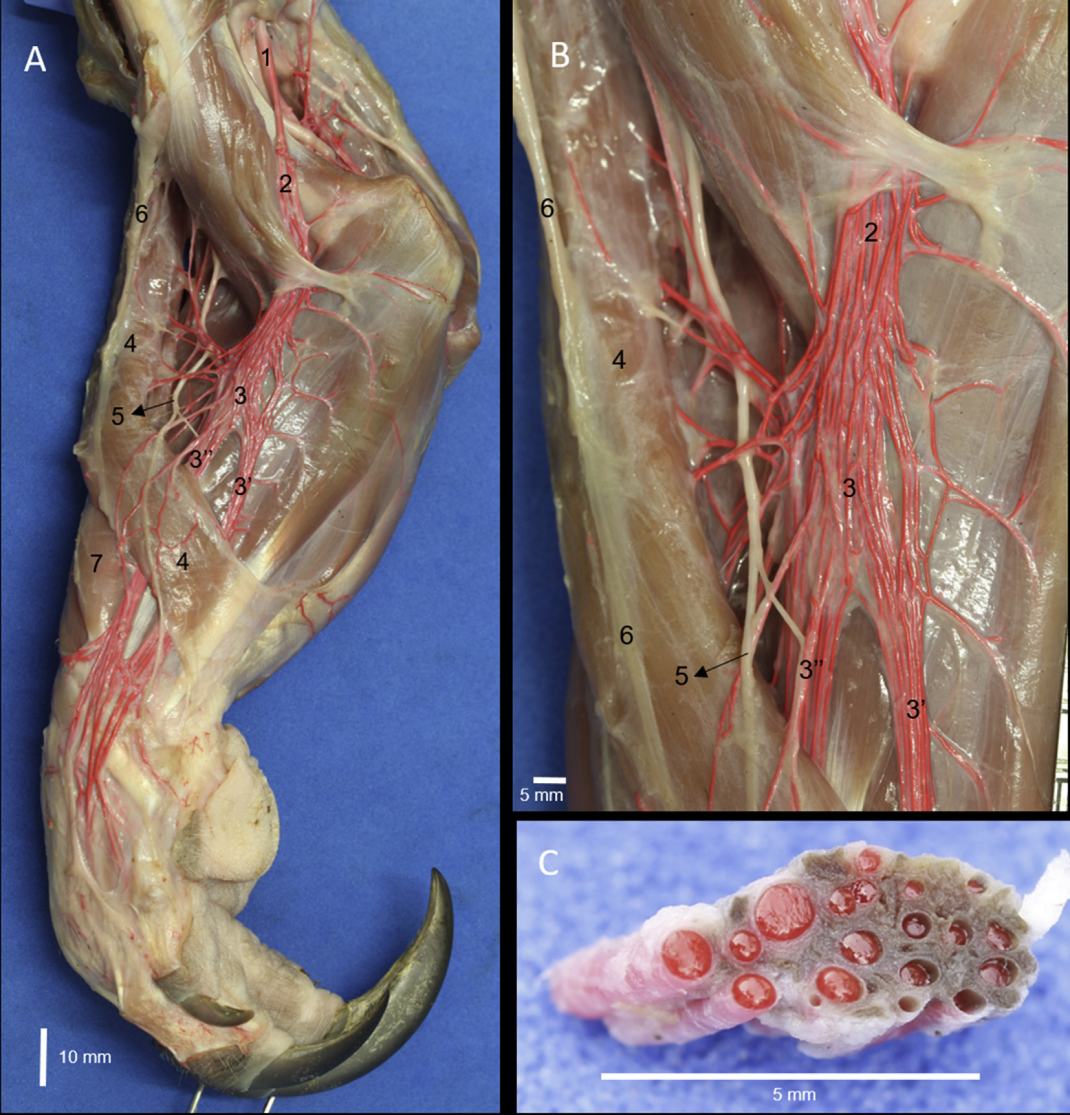
Arterial plexus (rete mirabile) from the superficial brachial artery. A) medial view of the right forearm, B) medial view of the proximal extreme of the forearm, C) cross section of the superficial brachial vessels (arteries: red; veins: dark). 1) brachial artery, 2) superficial brachial artery, 3) cranial superficial antebrachial artery, 3′) medial branch, 3″) lateral branch, 4) m. brachioradialis accesorius, 5) medial antebrachial cutaneus nerve, 6) cephalic vein, 7) m. brachioradialis.

The **m. brachioradialis accessorius** is a fusiform and flattened muscle. It has an origin in the distal part of the deltoid tuberosity, brachial fascia and deltoideus muscle (Fig. 1). Its belly is directed medially and is inserted onto the distal part of the antebrachial fascia and flexor retinaculum through a wide tendon. In this retinaculum, one sesamoid bone is observed that articulates synovially with the radial carpal bone (Figs. 2 and 3). It is innervated by the deep branch of the radial nerve and its arterial supply is by the superficial brachial artery and the medial and lateral branches of the cranial superficial antebrachial artery (Fig. 4). In terms of anatomical relation this muscle covers an arterial plexus that corresponds to the cranial superficial antebrachial artery; this muscle also covers the distal half of the m. pronator teres, m. flexor carpi radialis, median nerve and artery; superficial to this muscle pass the cephalic vein and the medial antebrachial cutaneous nerve (N. musculocutaneous) (Fig. 5).

The **m. brachioradialis** (*M. brachioradialis*) is fusiform and flattened, which has an origin in the most distal part of the deltoid tuberosity, distal to m. brachioradialis accessorius (Fig. 1). It presents a short tendon that is developed just proximal to its insertion onto the styloid process of the radius. It is innervated by the deep branch of the radial nerve and irrigated by the superficial brachial artery and the lateral branch of the cranial superficial antebrachial artery (Figs. 4 and 5). Regarding anatomical relations, at the distal end of this muscle, the accessory cephalic vein passes superficially, which is joined medially with the cephalic vein passing superficially along the m. brachioradialis accessories (Fig. 5). The superficial branch of the radial nerve passes proximally between the m. brachioradialis and the m. extensor carpi radialis.

**Fig. 6:**
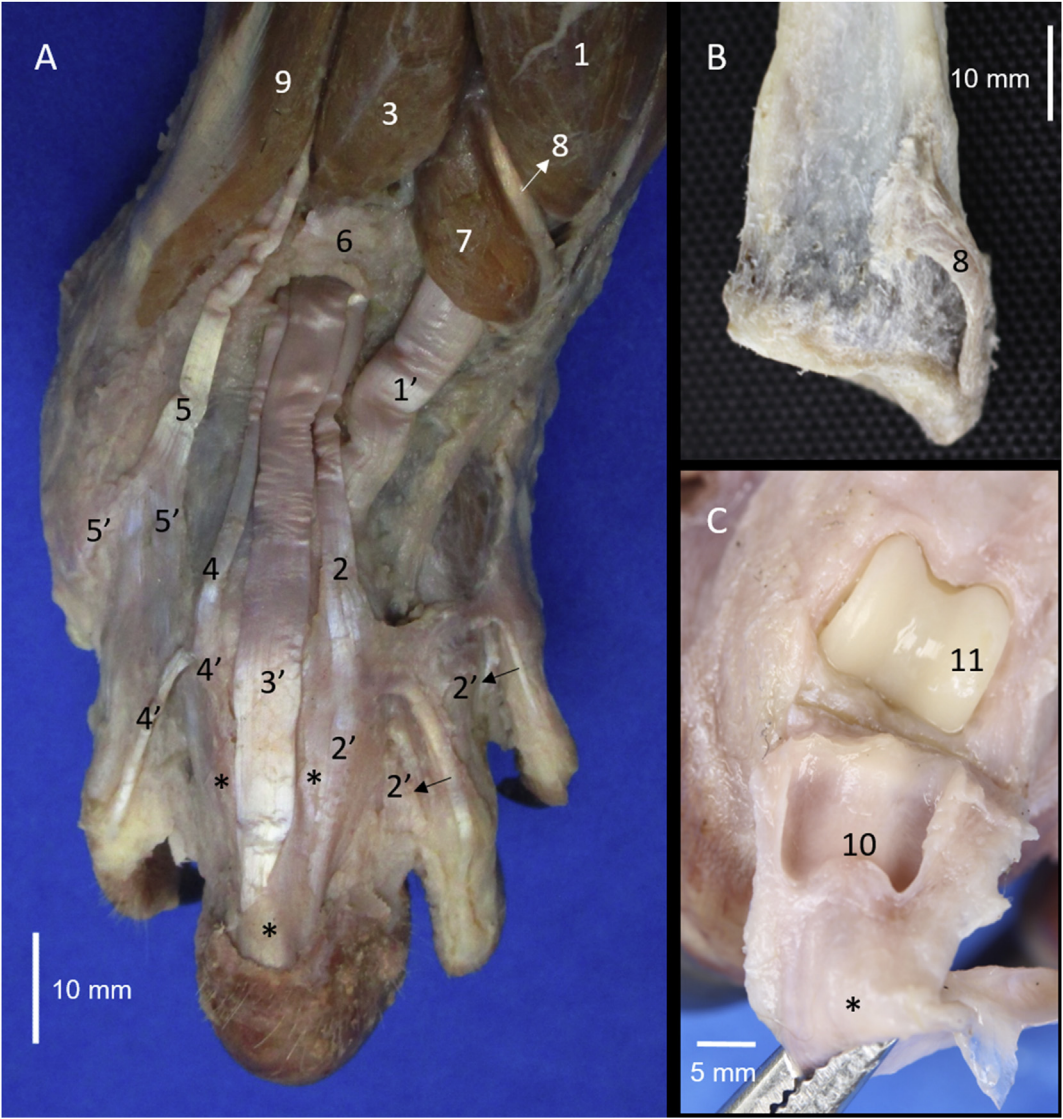
A) Dorsal view of the right hand. B) distal extreme of the radius (cranial view). C) dorsal view of the distal interphalangeal joint. 1) m. extensor carpi radialis, 2) m. extensor digiti I et II, 3) m. extensor digitorum communis, 4) m. extensor digiti III et IV, 5) m. extensor digitorum lateralis, 6) extensor retinaculum, 7) m. abductor longus I digiti, 8) retinaculum of the m. extensor carpi radialis, 9) m. extensor carpi ulnaris, 10) sesamoid cartilage, 11) head of the middle phalanx of the digit III. * extensor sheath for the tendon of the m. extensor digitorum communis. 1′-5′) tendons of the respective muscle.

**Fig. 7:**
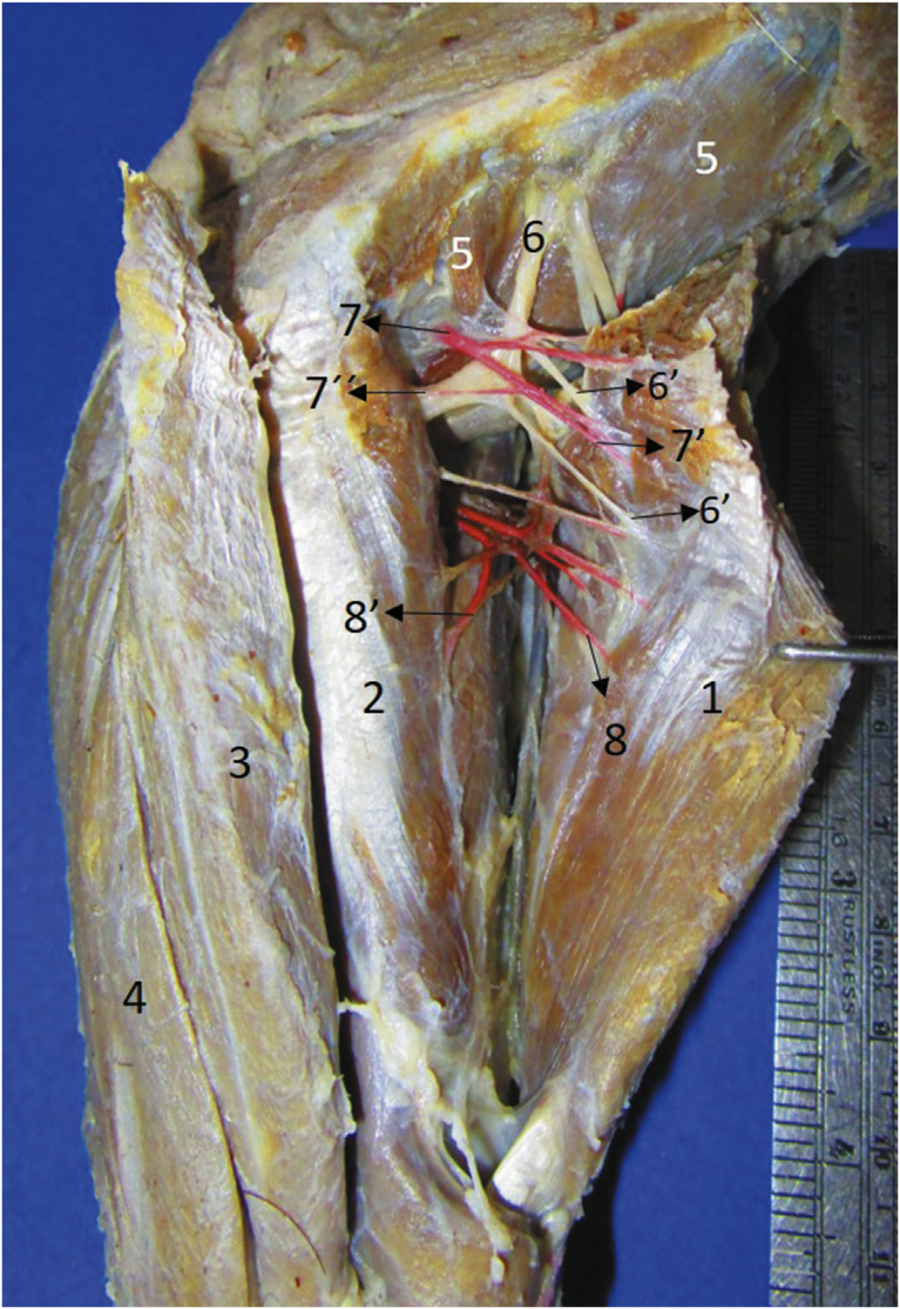
1) Extensor carpi radialis, 2) supinator, 3) extensor digitorum communis, 4) extensor carpi ulnaris, 5) m. brachialis, 6) Radial nerve, 6′) branches to 1, 7) transverse cubital artery, 8) superficial brachial artery, 8′) branch to 2.

The **m. extensor carpi radialis** (*M. extensor carpi radialis*) is a fusiform muscle. It has a fleshy and tendinous origin from the distal part of the deltoid tuberosity, deltoepicondylar ligament, the craniolateral surface of the lateral supracondylar crest of the humerus and the intermuscular septum with the m. extensor digitorum communis (Fig. 1). It develops a tendon just proximal to the tendon of the m. abductor digiti I longus, and deep of the latter is bridged by a retinaculum that goes from the distal part of the lateral margin of the radius until the styloid process of the radius (Figs. 2, 6A, 6B). The tendon inserts onto the dorsal part of the base of the metacarpal bone III. It is innervated by the deep branch of the radial nerve and its arterial supply is via the a. transverse cubital, a. superficial brachial, branch lateral of the a. cranial superficial antebrachial and one branch of the deep brachial artery when it passes together with the radial nerve (Fig. 7).

**Fig. 8:**
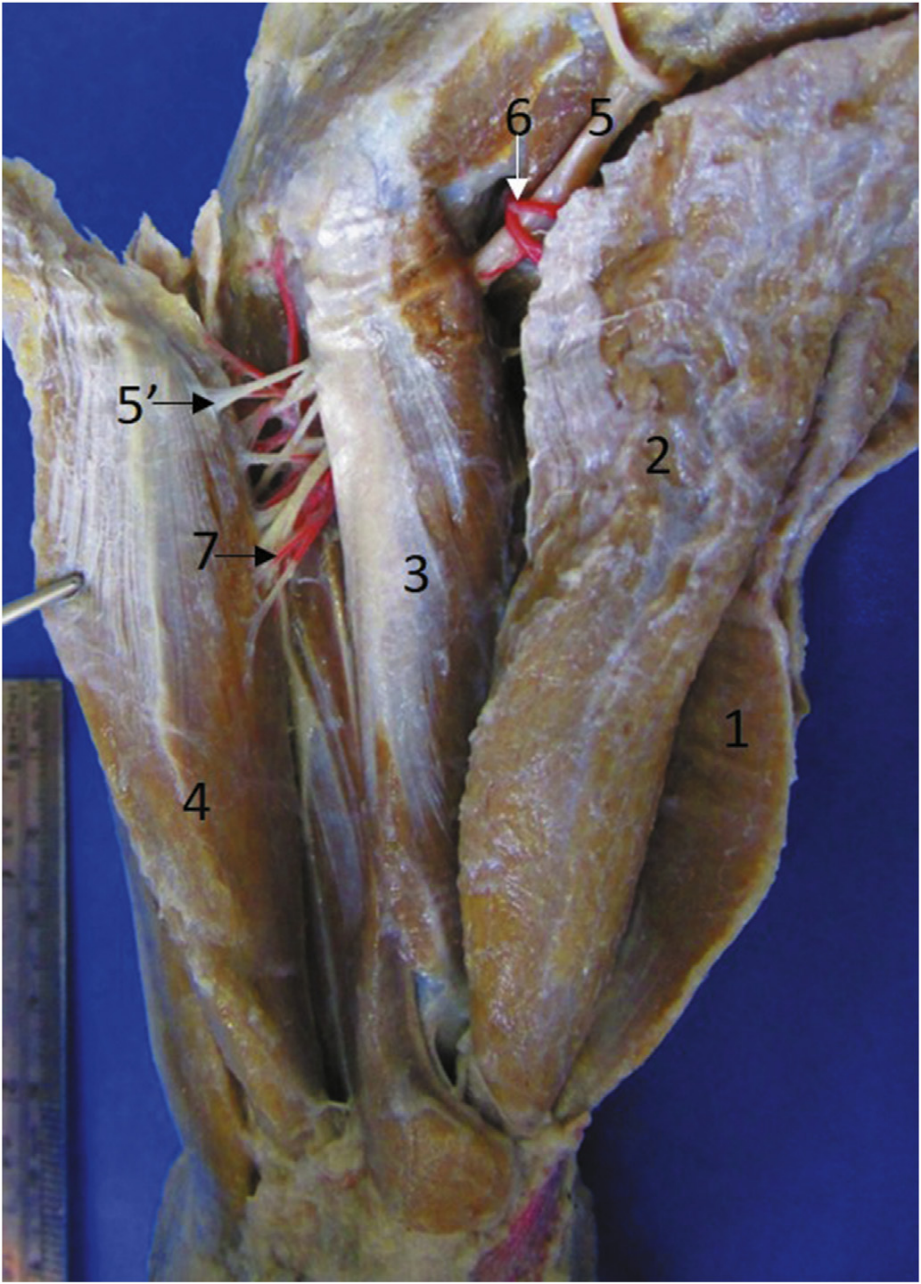
1) Brachioradialis, 2) extensor carpi radialis, 3) supinator, 4) extensor digitorum communis, 5) radial nerve, 5′) branch to 4, 6) transverse cubital artery, 7) cranial interosseous artery.

The **m. extensor digitorum communis** (*M. extensor digitorum communis*) in its proximal part is fusiform and in its distal part bipennate. It has a fleshy and tendinous origin in the lateral epicondyle of the humerus, antebrachial fascia, and intermuscular septum with the m. extensor carpi radialis in the proximal half of the forearm (Fig. 1). It develops a strong tendon proximal to the extensor retinaculum that goes independent and inserts onto the dorsal part of the base of the distal phalanx of digit III (Figs. 1, 6A). A synovial sheath surrounds the tendon at the level of the dorsal part of the metacarpal bone III until its insertion (Figs. 1 and 2). This synovial sheath is fused with the articular capsule of the digits' joints, which develops a dorsal sesamoid cartilage at the level of the dorsum. The more evident cartilage is in the distal interphalangeal joint of the digit III (Fig. 6C). This muscle is innervated by the deep branch of the radial nerve once it passes deep to the m. supinator, and its arterial supply is by the cranial interosseous artery (Fig. 8).

The **m. extensor digitorum lateralis** (*M. extensor digitorum lateralis*) is fusiform and has a fleshy and tendinous origin in the lateral epicondyle of the humerus, antebrachial fascia and intermuscular septum with the m. extensor carpi ulnaris (Fig. 1). Its fleshy belly is in the proximal half and distally developes a single tendon that passes through of the extensor retinaculum, and more distally, it bifurcates into a medial tendon that goes towards the digit IV to inserts onto the dorsal part of the base of the distal phalanx, and one lateral tendon that is inserted onto the base of the metacarpal bone V (Figs. 1 and 2). In one specimen the medial tendon is directed towards the sheath of the digit III. It is innervated by the deep branch of the radial nerve once it passes deep to the m. supinator, and its arterial supply is by the cranial interosseous artery.

The **m. extensor carpi ulnaris** (*M. extensor carpi ulnaris*) is fusiform and has a fleshy and tendinous origin in the most distal part of the lateral epicondyle of the humerus. It has a short tendon that inserts onto the base of the metacarpal bone V, and others tendinous fibers are directed towards the fascia that covers the carpal pad (palmar fascia) (Figs. 1 and 6). It is innervated by the deep branch of the radial nerve once it passes deep to the m. supinator, and its arterial supply is by the cranial interosseous artery.

**Fig. 9:**
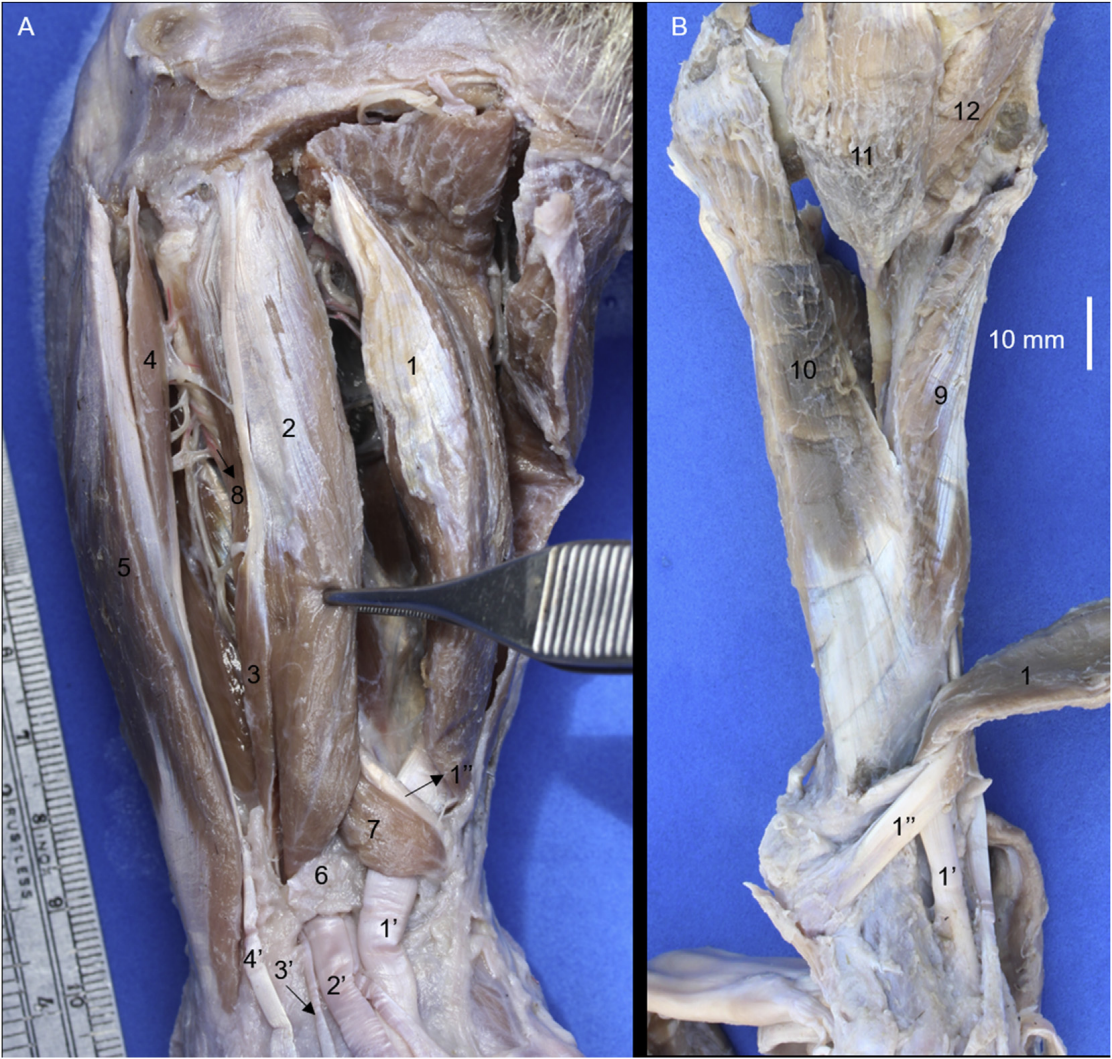
A) Craniolateral muscles of the right forearm B) cranial view of the supinator and pronator muscles in the left forearm. 1) m. extensor carpi radialis, 2) m. extensor digitorum communis, 3) m. extensor digiti III et IV, 4) m. extensor digitorum lateralis, 5) m. extensor carpi ulnaris, 6) extensor retinaculum, 7) m. abductor longus I digiti, 8) cranial interosseus arterial plexus, 9) m. supinator, 10) m. pronator teres, 11) m. bices brachi, 12) m. brachialis, 1′-5′) tendons of the respective muscle, 1″) retinaculum of the m. extensor carpi radialis.

The **m. extensor digiti III et IV** is a small and fusiform muscle that lies deep between the m. extensor digitorum communis and the m. extensor digitorum lateralis (Fig. 9). It has a tendinous origin from a common tendon with the m. extensor digitorum communis in the lateral epicondyle of the humerus. This tendon of origin is long and goes until the proximal half of the forearm, where a fleshy belly is developed, which sends one tendon that crosses the extensor retinaculum and just distal is bifurcated in a medial aponeurosis that is inserted onto the sheath of the tendon of m. extensor digitorum communis (digit III), and the other side, one lateral tendon inserts onto the base of the distal phalanx of digit IV (Figs. 2 and 6). In one specimen, the lateral tendon also sends an aponeurotic branch that covers the metacarpal bone V until inserting onto the base of the vestigial proximal phalanx. It is innervated by the deep branch of the radial nerve once it passes deep to the m. supinator, and its arterial supply is by the cranial interosseous artery.

The **m. supinator** (*M. supinator*) is fusiform and has a tendinous origin in the distal part of the lateral epicondyle of the humerus and lateral collateral ligament of the elbow and it inserts in a fleshy form in the three proximal quarters of the medial margin and the cranial aspect of the radius (Figs. 1, 2, 9). It is innervated by the deep branch of the radial nerve once it passes deep to the m. supinator and its arterial supply is by the cranial interosseous, cranial superficial antebrachial and cubital transverse arteries (Figs. 7, 8).

The **m. abductor digiti I longus** (*M. abductor digiti I longus*) is bipennate and has a fleshy origin along the lateral surface of the radius and the lateral surface of the ulna, intermuscular septum with the m. extensor digiti I et II and interosseous ligament. It inserts in a tendinous form onto the lateral part of the carpal bone I (Figs. 1 and 2). It is innervated by the deep branch of the radial nerve once it passes deep to the m. supinator, and its arterial supply is by the cranial interosseous artery.

The **m. extensor digiti I et II** (*M. extensor digit I, M. extensor digiti II*) is bipennate and has a fleshy origin in the caudal most part of the lateral surface of the ulna and the epimysium of the m. abductor digiti I longus (Fig. 1B). Its tendon is developed in the distal third of the forearm and goes free of muscle fibers when passing through the extensor retinaculum. Once it crosses the latter it divides into three tendons: one medial had an aponeurotic form, which inserts onto the base of the distal phalanx of the digit I; one intermediate inserts onto the base of the distal phalanx of digit II; and one lateral is joined to the sheath of the m. extensor digitorum communis, which inserts onto the deep part of the base of the distal phalanx of digit III (Fig. 2). It is innervated by the deep branch of the radial nerve and its arterial supply is by the cranial and caudal interosseous arteries.

### 3.2 Development of rete mirabile in the vessels for the craniolateral forearm muscles

The superficial brachial, cubital transverse, cranial and caudal interosseous arteries have a shape of rete mirabile sending branches for the craniolateral forearm muscles. The superficial brachial artery is formed from the brachial artery in the middle part of the arm and passed distally deep to the aponeurosis bicipital between the biceps brachii and pronator teres muscle, where it sends branches to both brachioradialis and extensor carpi radialis muscles. This artery continues distally as cranial superficial antebrachial artery, which is divided in two branches, lateral and medial, which also presents a shape of rete mirabile, and the distal extreme of the both forearm arterial plexus are anastomosed to direct towards the dorsum of the hand (Fig. 5). The transverse cubital artery is formed from the brachial artery just distal to the supracondylar foramen and passes towards lateral deep between the brachialis muscle and the humerus to reach the muscles of the forearm. The cranial and caudal interosseous arteries are formed from the common interosseous artery, which has shape of rete mirabile. The first passes among the interosseous antebrachial space towards craniolateral forearm muscles; and the second is present in the surface caudal of the interosseous ligament and sends perforating branches towards extensor digiti I et II muscle. All arterial plexuses are surrounded by a venous plexus (Fig. 5C).

### 3.3 Statistical analysis of the muscular weights

According to the Kruskal Wallis test the samples are not of the same population, and according to Dunn's multiple comparison test, statistical differences (α = 0.05) between the weights of the functional groups in the craniolateral forearm muscles are found among carpal extensors and digital extensors, even between supinators and digital extensors, but no statistical differences are found between carpal extensors and supinators (Table 3).

## 4 Discussion

### 4.1 Comparative myology

#### 4.1.1 M. brachioradialis

In *Tamandua* sp. a single m. brachioradialis has been described with two heads, one superficial and one deep but the author represents it graphically with a single origin in the deltoid tuberosity (Taylor, 1978). This is different to *T. mexicana* where two origins were observed independently for each brachioradialis muscle (Fig. 2), which has been described in *Tamandua tetradactyla*, but it is reported as a single muscle with two parts, one superficial and one deep (Humphry, 1869). However, although the reported insertion (Taylor, 1978; Humphry, 1869) is similar to that found in *T. mexicana*, we considered them as independent muscles because their origins, insertions, innervations and arterial supplies were independent, and besides that, the two muscles were superficial and their position goes in a collateral form, where the lateral is directed for the radius and the medial (m. brachioradialis accesorius) for the caudomedial part of the forearm where it covers part of the flexor muscles. On the other hand, Humphry (1869) also reports it inserted onto a supernumerary bone, which must correspond to the sesamoid bone of the flexor retinaculum found in *T. mexicana* articulating medially with the radial carpal bone. In *Myrmecophaga tridactyla* is reported that it can be bilaminate, with a superficial part and a deep part, with insertions similar to *Tamandua* sp. but with origin in the lateral supracondylar crest of the humerus (Pouchet, 1874; Macalister, 1875a). In *Cyclopes didactylus* it is described to originate in the deltoepicondilar ligament and is divided in two strata (Galton, 1869a), which are inserted similarly to *Tamandua* and *Myrmecophaga.*

In *Bradypus* sp*.* the brachioradialis muscle may be absent (Macalister, 1869), but when is present, its origin is in the lateral supracondylar crest of the humerus but with different dispositions, from a simple muscle (Macalister, 1869; Cuvier and Laurillard, 1850; Diniz et al., 2018; Olson et al., 2018) until divided in two parts but both inserted onto the radius (Humphry, 1869; Meckel, 1825; Mackintosh, 1870); or also attached to m. pronator teres proximal to its insertion (Cuvier and Laurillard, 1850) differing from *T. mexicana*. The last two dispositions can be present in two-digit sloths *Choloepus* sp*.* (Mackintosh, 1875; Windle and Parsons, 1899), although in this species (Humphry, 1869) it can be found with insertions similar to *T. mexicana*. The m. brachioradialis is absent in armadillos (Cuvier and Laurillard, 1850; Galton, 1869b; Macalister, 1875b; Windle and Parsons, 1899; Olson et al., 2015). In primates such as the human, the m. brachioradialis can have one accessory head as an anatomical variant (Standring, 2016; Testut and Latarjet, 1983), similar to *Saguinus leucopus* (Vélez-García et al., 2015) and *Saimiri sciureus* (Dunlap et al., 1985). However, in these species, when the accessory head is present, it joins with the insertion tendon, acting as a head and not as an independent muscle as in *T. mexicana.*

In domestic carnivores there is only one m. brachioradialis (Liebich et al., 2005; Dyce et al., 2012; Barone, 2000). In the dog it is less developed and it may even be absent, but when present it is a thin lamina, which originates in the lateral supracondylar crest of the humerus (Budras et al., 2007; Hermanson, 2013; Clair, 1982), different to the cat where it has a greater development and its origin is variant, from the craniolateral part of the middle third of the humerus (De Iuliis and Pulerà, 2006), or in the lateral supracondylar crest of the humerus, but in both species it is inserted proximally to the styloid process (Dyce et al., 2012; Liebich et al., 2005; Clair, 1982; Barone, 2000), being similar to m. brachioradialis of *T. mexicana*, but in this species there are two brachioradialis muscles with a different origin and both highly developed.

#### 4.1.2 M. extensor carpi radialis

The m. extensor carpi radialis of *T. mexicana* had an origin and insertion similar to what is described in *Tamandua* sp*.* (Taylor, 1978), however this author does not report an origin in the intermuscular septum with the m. extensor digitorum communis. In *T. tetradactyla* and *M. tridactyla* the origin is not specified but its insertion was similar (Windle and Parsons, 1899; Rapp, 1852; Macalister, 1875a) to *T. mexicana*. In *C. didactylus*, this muscle has a similar disposition to *T. mexicana* (Galton, 1869a, Humphry, 1869, Macalister, 1875a,b).

In others xenarthrans species such as the *Ch. didactylus* and *B. tridactylus*, the m. extensor carpi radialis is originated from the lateral epicondyle of the humerus and its insertion occurs through two tendons, one is inserted onto the metacarpal bone II and the other more developed in the metacarpal bone III (Mackintosh, 1875; Windle and Parsons, 1899; Humphry, 1869; Macalister, 1869), being different to *Tamandua* sp. and *M. tridactyla* (Taylor, 1978; Macalister, 1875a; our study). In *Ch. dydactilus* and *Ch. hoffmani* two extensor carpi radialis have been reported - one brevis and one longus, both originated from the supracondylar ridge of the humerus (Mendel, 1979, 1981), differing to other authors and other xenarthrans. In *Bradypus* sp. it is a single muscle and can be found with a single tendon inserted onto the metacarpal bone III (Windle and Parsons, 1899), metacarpal bone II (Mackintosh, 1870), or both (Olson et al., 2018), even onto the distal row of the carpus (Diniz et al., 2018).

The armadillo's species (Cuvier and Laurillard, 1850; Galton, 1868; Macalister, 1875a,b; Windle, 1875; Windle and Parsons, 1899; Olson et al., 2015) differ from *Tamandua mexicana* because this muscle is not originated from a deltoepicondilar ligament and also develops more than one tendon.

The m. extensor carpi radialis in the dog is divided into two tendons that are inserted onto the metacarpal bone II and III (Liebich et al., 2005; Budras et al., 2007; Hermanson, 2013). It also differs from the cat, since in this species there are two muscles, m. extensor carpi radialis longus and m. extensor carpi radialis brevis, where the longus is inserted onto the metacarpal bone II and the brevis onto the metacarpal bone III (Liebich et al., 2005; Barone, 2000; Clair, 1982).

#### 4.1.3 M. extensor digitorum communis

Taylor (1978) in his illustrations of the humerus of *Tamandua* sp*.* represents the origin of the m. extensor digitorum communis in the distal part of the lateral supracondylar crest of the humerus and the lateral epicondyle of the humerus, differing from our results in *T. mexicana* where we only observed it originated from the lateral epicondyle of the humerus. However, in *T. tetradactyla,* it is reported originated in the lateral epicondyle of the humerus and sends tendons for the digits III and IV (Windle and Parsons, 1899), differing from *T. mexicana* where it sends only one tendon for the digit III, since a separate muscle was observed that is directed for the digit III and IV. In *C. didactylus* it is originated from the lateral supracondylar crest of the humerus, distal to the m. extensor carpi radialis, with the m. abductor digiti I longus (Galton, 1869a, Humphry, 1869, Macalister, 1875a,b, Cuvier and Laurillard, 1850), which differs to *T. mexicana* but presents the same insertion. In *M. tridactyla* it is reported originated in the lateral epicondyle of the humerus and directed towards digits II, III and IV (Macalister, 1875a), although it can also send only for the digits III and IV (Windle and Parsons, 1899), or for all the digits (Pouchet, 1874), varying in the distribution of their tendons with *T. mexicana.*

In sloths this muscle also varies with respect to *T. mexicana*, since in *Ch. didactylus* it has an origin on the lateral epicondyle of the humerus and inserts onto the distal phalanx of the digits II and III (Mackintosh, 1870; Windle and Parsons, 1899), or sometimes also onto the base of the metacarpal bone IV (Mendel, 1981). In *B. tridactylus* this muscle has a similar origin to the species named above, but differs by being inserted onto the distal phalanx of the digits II, III and IV (Windle and Parsons, 1899; Humphry, 1869; Mackintosh, 1870; Cuvier and Laurillard, 1850; Diniz et al., 2018; Olson et al., 2018), or digits II and III (Macalister, 1875a).

In armadillos, the origin is similar to *T. mexicana*, but differs in the formation of tendons, as in *D. novemcinctus* that sends for the digits IV and V (Olson et al., 2015); or in *C. villosus* and *C. truncatus* their tendons are directed for digits II, III and IV (Windle and Parsons, 1899; Macalister, 1875b). In *E. sexcinctus* this muscle arises from the lateral supracondylar crest of the humerus and sends two tendons, one medial for digits II and III and one lateral for III and IV digits (Galton, 1869a,b; Cuvier and Laurillard, 1850), which also differs to *T. mexicana*, although this species has another muscle for III and IV.

In domestic carnivores, the origin of the m. extensor digitorum communis is in the lateral epicondyle of the humerus (Dyce et al., 2012; Liebich et al., 2005; Hermanson, 2013; Budras et al., 2007). However, in the cat, another author reports its origin from the lateral supracondylar crest of the humerus, distal to the origin of the m. extensor carpi radialis brevis (Sebastiani and Fishbeck, 2005), but in both species the tendons are inserted onto the extensor process of the distal phalanx of digits II, III, IV and V (Dyce et al., 2012; Liebich et al., 2005; Hermanson, 2013; Budras et al., 2007; Sebastiani and Fishbeck, 2005), differing from *T. mexicana* since in this species it is only directed for the digit III.

#### 4.1.4 M. extensor digitorum lateralis

The m. extensor digitorum lateralis in *Tamandua* sp. is not reported by Taylor (1978). The origin in *M. tridactyla* is in the lateral epicondyle of the humerus and is inserted onto the digits IV and V (Macalister, 1875a), however other authors report it inserted only onto the digit V (Windle and Parsons, 1899; Pouchet, 1874). In *T. tetradactyla* the insertion is only onto the digit V (Windle and Parsons, 1899), which differs from *T. mexicana* where digit V is not found, but is directed for the digit IV and the metacarpal bone V. Otherwise, in *C. didactylus* the muscle is originated from the lateral supracondylar crest of the humerus and adjacent muscles, and is inserted through a wide tendon at the base of the proximal phalanx of the digit III (Galton, 1869a), different to *T. mexicana*, where it goes mainly to digit IV, although it can go for digit III as an anatomical variant*.*

In *Bradypus* sp., it was reported with the origin in the lateral epicondyle of the humerus and inserted onto the base of metacarpal IV (Diniz et al., 2018; Olson et al., 2018), while in *Choloepus* sp. it was inserted onto the metacarpal III (Mendel, 1981). Old descriptions report that this muscle is replaced by the extensor digiti brevis muscles, which have the origin in the dorsum of the carpus and metacarpus, and is inserted in one or all the digits (Humphry, 1869; Macalister, 1869), however current studies demonstrate the presence of both muscles (Diniz et al., 2018; Olson et al., 2018), differing from Vermilingua.

In *D. novemcinctus,* the author names it as m. Abductor digiti V longus, which differs in its insertion to *T. mexicana*, since it is inserted onto the metacarpal bone V (Olson et al., 2015). In *E. sexcinctus* the muscle is small and flat and has its origin in the most distal part of the lateral supracondylar crest of the humerus, and its insertion is similar to that of the species previously named (Galton, 1869b). On the other hand, in *C. villosus* and *C. truncatus* the muscle is inserted in digits IV and V (Windle and Parsons, 1899; Macalister, 1875b), presenting a similar insertion with *T. mexicana*.

This muscle in the domestic dog originates from the lateral epicondyle of the humerus, with additional fibers from the lateral collateral ligament of the elbow (Liebich et al., 2005; Hermanson, 2013; Budras et al., 2007). It also forms two tendons, one lateral that is inserted onto the proximal phalanx of digit V, while the medial tendon bifurcates and inserts onto the proximal phalanx of digits III and IV (Liebich et al., 2005; Hermanson, 2013; Budras et al., 2007). The origin in *F. catus* is in the lateral supracondylar crest of the humerus and is inserted through three tendons onto the digits III to V (Liebich et al., 2005; Sebastiani and Fishbeck, 2005), or through four tendons that are directed to each principal digit (Clair, 1982), differing from *T. mexicana* since in this species it is mainly directed for the digit IV.

#### 4.1.5 M. extensor carpi ulnaris

Taylor (1978) in his humeral graphics of *Tamandua sp.* represents the origin of the m. extensor carpi ulnaris in the most distal part of the lateral epicondyle of the humerus, being similar to *T. mexicana*, however, the author does not specify its insertion. In *M. tridactyla* and *T. tetradactyla* the muscle is simple with an origin in the most distal part of the lateral epicondyle of the humerus and inserts onto the metacarpal bone V (Macalister, 1875a; Windle and Parsons, 1899), thus the disposition of this muscle in these two species is similar to that found in *T. mexicana*. However, the insertion onto the palmar fascia is not reported in either of these species. On the other hand, in *C. didactylus* there are two muscles, both with origin on the lateral epicondyle of the humerus, the largest is inserted onto the metacarpal bone III and the other that has a slight adhesion to the ulna, is inserted onto the metacarpal bone V rudimentary (Humphry, 1869), differing to *T. mexicana*.

In *Choloepus*, it has an origin in the olecranon, the body of the ulna and the lateral epicondyle of the humerus, and is divided into two parts, which are inserted in a tendinous form onto the dorsal aspect of the proximal phalanx of digit III and in the base of the metacarpal bone IV (Mackintosh, 1875; Humphry, 1869; Mendel, 1979, 1981), thus differing to *T. mexicana* in both the origin and the insertion. On the other hand, in *B. tridactylus* there are two muscles, one with tendinous origin in the lateral epicondyle of the humerus and that is inserted onto the distal part of the metacarpal bone IV, while the other muscle has an origin in the lateral part of the ulna and is inserted onto the base of the metacarpal bone V (Humphry, 1869). Other authors report the presence of a single muscle, which arises from the lateral epicondyle of the humerus and is inserted onto the metacarpal bone III (Macalister, 1869; Windle and Parsons, 1899; Mackintosh, 1870; Diniz et al., 2018), or metacarpal bone IV (Olson et al., 2018), presenting a different disposition to *T. mexicana*, since in this species we find it directed to metacarpal bone V and palmar fascia.

In armadillos, the origin varies widely to *T. mexicana*, since in *D. novemcinctus* the origin is in lateral margin of the ulna and from the intermuscular septum with the m. extensor digitorum lateralis (Olson et al., 2015); and in *E. sexcinctus* the origin is in the lateral supracondylar crest of the humerus and fuses at its origin with fibers of the adjacent muscle (Galton, 1869b). In *C. villosus* and *C. truncatus* it originates in the lateral epicondyle of the humerus (Windle and Parsons, 1899; Macalister, 1875b), but in all species as *T. mexicana*, it is inserted onto the base of metacarpal bone V (Olson et al., 2015; Galton, 1869a,b; Windle and Parsons, 1899; Macalister, 1875a).

The origin and insertion of this muscle in *T. mexicana* is similar to domestic carnivores (Hermanson, 2013; Budras et al., 2007; Liebich et al., 2005; Sebastiani and Fishbeck, 2005), however, it can differ to the domestic dog, where it can also be inserted onto the accessory carpal bone (Dyce et al., 2012).

#### 4.1.6 M. extensor digiti III et IV

The m. extensor digiti III et IV is only present is absent in the other cited xenarthrans (Humphry, 1869; Macalister, 1869; Galton, 1869a,b; Mackintosh, 1870; Macalister, 1875a,b; Mackintosh, 1875; Windle and Parsons, 1899; Olson et al., 2015) and domestic carnivores (Budras et al., 2007; Dyce et al., 2012; Hermanson, 2013). Therefore, this muscle might be fused to the m. extensor digitorum communis in the other species.

#### 4.1.7 M. supinator

The origin of the m. supinator in the collateral lateral ligament of the elbow is not reported by Taylor (1978), differing from our findings in *T. mexicana*. In *M. tridactyla* it is inserted along the entire length of the medial margin of the radius (Macalister, 1875a), however other authors find it inserted onto the most distal part of the radius (Windle and Parsons, 1899; Pouchet, 1874). In *T. tetradactyla* the insertion is similar to *M. tridactyla* (Windle and Parsons, 1899), which differs to *T. mexicana* since in this species the fourth distal of the medial margin of the radius is free of insertion. In *C. didactylus* it is a well-developed muscle, arising from the lateral supracondylar crest of the humerus, and inserted along the medial margin of the radius (Galton, 1869a), similar to *M. tridactyla* but different of *T. mexicana*.

In *Ch. didactylus* it is a large muscle and is divided in two layers (Windle and Parsons, 1899), however Humphry (1869) did not find this division, and reported that it is inserted in the proximal third of the radius (Mackintosh, 1875). In *B. tridactylus* it is a small muscle (Macalister, 1869), but the origin and insertion are similar to *Choloepus* sp. (Diniz et al., 2018; Olson et al., 2018).

In armadillos such as *D. novemcinctus,* this muscle is absent (Olson et al., 2015) or could be present as a very small muscle in *E. sexcinctus* (Galton, 1869b), *C. villosus* (Windle and Parsons, 1899) and in *C. truncatus* (Macalister, 1875a,b), differing to vermilinguas and sloths where it is very well developed.

In domestic carnivores is a fusiform muscle, where the origin is similar to *T. mexicana* but is only inserted onto the proximal third of the medial margin of the radius (Liebich et al., 2005; Hermanson, 2013; Dyce et al., 2012; Budras et al., 2007).

#### 4.1.8 M. abductor digiti I longus

Taylor (1978) in his graphic of radius and ulna of *Tamandua* sp. represents the origin of the m. abductor digiti I longus similar to *T. mexicana* but does not report the origin from the interosseous ligament. In *M. tridactyla* the origin is from a crest in the proximal and caudal part of the ulna and inserts onto the base of the metacarpal bone I (Macalister, 1875a), different to *T. mexicana*, where it is inserted onto the carpal bone I. In *T. tetradactyla* the authors do not specify the origin and insertion of the muscle (Windle and Parsons, 1899). In *C. didactylus* the origin is in the lateral supracondilar crest of the humerus and is inserted by a strong tendon in the rudimentary I digit (Galton, 1869a).

In sloths, the radial origin and interosseous ligament are not found, and otherwise, their insertion varies, since in *Choloepus* they are inserted onto the carpal bone I (Mackintosh, 1875; Mendel, 1981) similar to *T. mexicana*, but different from *Bradypus* sp., where it is inserted onto the base of metacarpal bone I (Macalister, 1869; Mackintosh, 1870; Diniz et al., 2018; Olson et al., 2018), or also partially onto metacarpal bone II (Olson et al., 2018).

In *D. novemcinctus* there is no radial origin and it is inserted onto the metacarpal bone II due to the loss of the digit I (Olson et al., 2015), differing to *T. mexicana* where there is a digit I, however the insertion is onto carpal bone I. In *E. sexcinctus* it is a large muscle and the origin is similar to that of the species named previously, but it is inserted at the base of metacarpal bone I (Galton, 1869b), the same as in *C. villosus* (Windle and Parsons, 1899) and in *C. truncatus* (Macalister, 1875b), differing both in the origin and insertion to *T. mexicana*.

In domestic carnivores, it is originated from the radius and the ulna, and is inserted onto the *Os sesamoideum m. abductor digiti I longi* and the metacarpal bone I (Hermanson, 2013; Liebich et al., 2005; Budras et al., 2007; Sebastiani and Fishbeck, 2005) differing to *T. mexicana.*

#### 4.1.9 M. extensor digiti I et II

Taylor (1978) in his graphics of radius and ulna of *Tamandua* sp. represents the origin of the m. extensor digiti I et II similar to *T. mexicana* (our findings) and *T. tetradactyla* (Windle and Parsons, 1899; Rapp, 1852). In *M. tridactyla,* it originates from the caudal part of the ulna and sends a thin tendon to the digit I, which is attached to the fascicle of the m. extensor digitorum communis (Macalister, 1875a), however, it also sends a tendon for the digit II (Windle and Parsons, 1899). Otherwise, in *C. didactylus* its origin is in the distal part of the ulna and on the dorsal surface of the carpal bones II and IV to send tendons to the digits II and III (Galton, 1869a,b, Humphry, 1869, Macalister, 1875a), differing in the origin and distribution of tendons to *T. mexicana*.

In *Bradypus* sp*.* it is a small muscle, arises from the most distal end of the ulna and inserts at the base of the proximal phalanx of digit I (Macalister, 1869; Mackintosh, 1870), middle phalanx of digit II (Olson et al., 2018), or metacarpal bone II (Diniz et al., 2018). In *Choloepus* sp*.* the muscle extends from the middle of the ulna to the rudimentary digit II (Mackintosh, 1875), or can sent a tendon variably to the metacarpal bone III (Mendel, 1981), differing in origin and insertion to *T. mexicana*.

In armadillos such as *D. novemcinctus,* its origin is in a small area of the lateral surface of the olecranon and only goes to the digit III (Olson et al., 2015). In *E. sexcinctus* it is a thin and flat muscle, arises from the lateral supracondylar crest of the humerus and extends along the caudal margin of the ulna (Galton, 1869b). Its tendon is divided into two parts, one wide and flat that is inserted onto the base of the proximal phalanx of digit II and another thin part that is inserted at the base of the distal phalanx of digit I (Galton, 1869b). In *C. villosus,* the distribution of the tendons is similar to that of the species previously named (Windle and Parsons, 1899), while in *C. truncatus* it arises near the olecranon and its tendon goes only to the digit I (Macalister, 1875a,b). Therefore, the distribution of the tendons in these species of armadillos differs to *T. mexicana* since in this species the tendons are directed for the digits I, II and III.

In domestic carnivores such as the dog it is an extremely small, thin and flat muscle, that is originated in the middle third of the caudolateral margin of the ulna (Liebich et al., 2005; Hermanson, 2013; Budras et al., 2007). Its tendon is divided in two, the medial is directed to the metacarpal bone I and the lateral one is directed to the digit II that joins the tendon of the m. extensor digitorum communis (Liebich et al., 2005; Hermanson, 2013; Budras et al., 2007). In *F. catus*, it is more developed than in the dog (Clair, 1982) and originates in the caudo-lateral margin of the ulna and its tendon is divided in three, the medial goes to digit I, and the other two tendons are go to the digit II (Liebich et al., 2005; Sebastiani and Fishbeck, 2005), differing in the distribution of the tendons to *T. mexicana* since normally in this species, it sends tendon for the digit III. However in the dog, the development of a tendon for this digit can occur variably (Clair, 1982; Hermanson, 2013), similar to another wild canid such as the crab-eating fox (*Cerdocyon thous*) (García et al., 2015).

### 4.2 Comparative nerve and arterial supply

The innervation for the craniolateral muscles of the forearm in *T. mexicana* was similar to that reported in domestic carnivores, where only the deep branch of the radial nerve participates (Budras et al., 2007; Dyce et al., 2012; Hermanson, 2013; Evans and De Lahunta, 2013). The only difference in *T. mexicana* was that one muscular branch was bifurcated to send one branch for each brachioradialis muscle (Fig. 4). The nerve passes deep to the m. supinator as occurs in the dog (Barone, 2000; Budras et al., 2007; Evans and De Lahunta, 2013).

Arterial supply of the craniolateral muscles of the forearm of *T. mexicana* has a significant variation with respect to that reported in domestic carnivores, since arterial plexuses are not formed in these species as we found it in *T. mexicana*; however by comparative anatomy the following differences were found in terms of arterial supply for these muscles: In the dog, the cubital transverse artery supply to the m. extensor digitorum communis (Bezuidenhout, 2013), besides supplying the other extensor muscles of the carpus and digits (Ghoshal, 1982), differing *T. mexicana* where the cubital transverse artery only supplies the m. extensor carpi radialis and m. supinator. Otherwise, in the domestic dog, the caudal interosseous artery sends branches to the m. extensor digitorum communis, extensor digitorum lateralis, m. abductor digiti I longus and m. extensor digiti I et II (Ghoshal, 1982; Bezuidenhout, 2013), where the latter muscle in *T. mexicana* is the only one supplied by that artery. Barone (2000) in domestic carnivores reports the irrigation of the m. supinator by branches of the collateral radial artery or the median artery (depending on the species), differing from *T. mexicana* where this muscle receives its arterial supply from the cranial interosseous artery, superficial brachial artery and cubital transverse artery. The arterial supply by the cranial interosseous artery for these muscles in *T. mexicana* is consistent with that reported in domestic carnivores (Ghoshal, 1982; Barone, 2000; Bezuidenhout, 2013).

### 4.3 Functional analysis

According to the anatomical disposition of the m. brachioradialis in *T. mexicana*, it should act as an elbow flexor and a supinator of the forearm and the hand, which are functions reinforced by the m. brachioradialis accessorium, agreeing with Taylor (1978), however, the insertion onto flexor retinaculum of the latter muscle must generate tension into this retinaculum and at the same time increase the space of the carpal canal to allow a better sliding of the tendon of the m. flexor digitorum profundus and also to avoid the compression of the median nerve and artery, similar to the proposed function of the m. palmaris longus in primates (Vélez-García et al., 2018). In sloths, the brachioradialis muscle is large (Diniz et al., 2018; Olson et al., 2018), it even represents a great weight among the thoracic limb musculature, therefore it has been indicated as a strong elbow flexor (Olson et al., 2018), being different to *T. mexicana*, where it is a simple complementary muscle to the elbow flexion.

The m. supinator increases the strength of supination to the brachioradialis muscles in *T. mexicana*, since it has a wide insertion onto the radius, being similar to other species of the suborder Vermilingua (Pouchet, 1874; Galton, 1869a,b; Macalister, 1875a,b; Windle and Parsons, 1899; Taylor, 1978), but differing from other species where the strength of supination is lower because the insertion of the m. supinator is more proximal such as in sloths (Macalister, 1869; Mackintosh, 1875; Diniz et al., 2018) and domestic carnivores (Liebich et al., 2005; Sebastiani and Fishbeck, 2005; Dyce et al., 2012; Hermanson, 2013). However, in sloths this function is performed with more strength by the brachioradialis muscle (Olson et al., 2018). These functions of the brachioradialis, brachioradialis accessorius and supinator muscles are indispensable in *T. mexicana*, since when it walks quadripedally and its weight is supported on the side of its hands in supination (Yalden, 1972; Orr, 2005), similar to *T. tetradactyla* (Machado-Cruvinel et al., 2019). This species cannot support its palm completely into substrate due to the digit III and the greater development of the claws; therefore, this anatomical disposition interferes in the middle of the palm. The supination is unnecessary in armadillos, since they do not have these muscles (Olson et al., 2015) or if the m. supinator is present, this is vestigial (Macalister, 1875a,b), which is due to its digging habits.

The m. extensor carpi radialis in *T. mexicana* is not only an extensor of carpus and adductor of the hand (medial deviation), but an elbow flexor due to its more proximal origin in the humerus, which are functions complemented by the brachioradialis muscles, agreeing with Taylor (1978), and with a similar function to *C. didactylus* (Galton, 1869a; Humphry, 1869; Macalister, 1875a). These functions are performed with greater force in the Vermilingua suborder, since in other xenarthrans such as sloths (Galton, 1869a,b; Macalister, 1869; Mackintosh, 1870; Macalister, 1875a,b; Windle and Parsons, 1899; Mendel, 1981; Diniz et al., 2018; Olson et al., 2018) and armadillos (Galton, 1869a,b; Macalister, 1875a,b; Windle and Parsons, 1899; Olson et al., 2015), and even in domestic carnivores (Clair, 1982; Budras et al., 2007; Dyce et al., 2012; Hermanson, 2013), the origin of this muscle is more distal from the lateral supracondylar crest and the lateral epicondyle of the humerus, therefore, in these species its participation in elbow flexion is lower. The presence of a strong retinaculum in the radius that protects its tendon, deep to the m. abductor digiti I longus, corroborates its high development and its important function in the craniolateral forearm musculature in *T. mexicana*. This retinaculum apparently was reported by Macalister (1875a) in *M. tridactyla* and *C. didactylus*, since he described that this muscle is bridged by a thin fibrous band. The markedly larger development of this muscle could explain the higher capacity of more extension of the carpus and more extended hand postures of *Tamandua* ​when compared with *Myrmecophaga* ​(Yalden, 1972; Orr, 2005).

The m. extensor carpi ulnaris in *T. mexicana* should be an extensor and abductor of the hand at the carpus (lateral deviation), in addition to generate tension into the fascia that covers the palmar pad, which should be necessary to give resistance and protect it while the hand is supported on the ground. The strength for the extension of the carpus is greater in *C. didactylus* since it has two heads, where one of these is originating from the ulna (Humphry, 1869). Its function as extensor of the carpus is also present in armadillos (Galton, 1869b; Macalister, 1875a,b; Windle and Parsons, 1899; Olson et al., 2015) and other anteaters and sloths (Humphry, 1869; Mackintosh, 1875; Mendel, 1981; Diniz et al., 2018; Olson et al., 2018), however in none of these species is the insertion onto the palmar fascia reported. In domestic carnivores, the muscle has a contrary function such as the flexion of the carpus (Hermanson, 2013), except in the domestic cat where it is reported as a carpal extensor (Sebastiani and Fishbeck, 2005), as it must be in *T. mexicana*.

The m. abductor digiti I longus in *T. mexicana* was inserted onto the carpal bone I and not onto the metacarpal bone I, therefore it must act as an extensor, adductor and lateral rotator of the carpus. It does not produce abduction of the digit I as its name describes, which occurs in domestic carnivores because their insertion is onto metacarpal bone I (Clair, 1982; Budras et al., 2007; Dyce et al., 2012; Hermanson, 2013). Although in other species, there are reports of the insertion onto the metacarpal bone, and the function should similar to *T. mexicana*, since their digiti I is absent and there are only vestigial bones in sloths (Macalister, 1869; Mackintosh, 1870; Macalister, 1875a; Windle and Parsons, 1899; Mendel, 1981; Diniz et al., 2018; Olson et al., 2018) and armadillos (Galton, 1869b; Macalister, 1875a,b; Windle and Parsons, 1899; Olson et al., 2015).

The extension of the digits in *T. mexicana* is concentrated in the extension of the digit III, because the m. extensor digitorum communis is highly developed and sends a strong independent tendon for this digit. Windle and Parsons (1899) report that in *T. tetradactyla,* this muscle sends tendons for the digits III and IV, and therefore, in this species it also extends the digit IV. However, this is controversial for our findings in *T. mexicana*, since the m. extensor digitorum communis extends only the digit III, and there is a muscle that extends the digits III and IV as an independent muscle. In *C. didactylus*, it is also only goes for the digit III (Galton, 1869a; Humphry, 1869; Macalister, 1875a), different to *M. tridactyla* where it goes for the digits II-IV (Macalister, 1875a), similar to sloths and armadillos where it is directed to more than one digit (Cuvier and Laurillard, 1850; Galton, 1869a,b; Humphry, 1869; Mackintosh, 1870; Macalister, 1875a; Windle and Parsons, 1899; Olson et al., 2015). In domestic carnivores it is also directed for the digits II-V (Liebich et al., 2005; Sebastiani and Fishbeck, 2005; Budras et al., 2007; Dyce et al., 2012; Hermanson, 2013), and although *T. mexicana* has four digits, this muscle is only directed for the digit III. In addition, this muscle is supported by the tendons of the other less developed muscles, which send tendons towards the sheath of the tendon of m. extensor digitorum communis. Thus, the other muscles such as the m. extensor digiti I et II and m. extensor digiti III et IV, are responsible for supporting the extension of the digit III, but through of the tension of sheath, which is also protected by the sesamoid cartilage located on the dorsal surface of the articular capsule of the distal interphalangeal joint.

The extension of the other digits in *T. mexicana* is through the m. extensor digiti I et II, m. extensor digiti III et IV, and m. extensor digitorum lateralis. The m. extensor digiti III et IV is absent in other species of vermilingua (Galton, 1869a; Humphry, 1869; Macalister, 1875a; Windle and Parsons, 1899), sloths (Macalister, 1869; Humphry, 1869; Mackintosh, 1870, 1875; Macalister, 1875a; Windle and Parsons, 1899) and armadillos (Galton, 1869b; Macalister, 1875a,b; Olson et al., 2015). However, its function is compensated by more tendons of the m. extensor digitorum communis, or in the case of sloths, it is compensated also by the presence of the extensor digitorum brevis muscles on the dorsum of the hand (Mackintosh, 1875; Windle and Parsons, 1899; Mendel, 1981; Diniz et al., 2018; Olson et al., 2018). In *T. mexicana* the second most developed digital extensor muscle is the m. extensor digiti I et II, which is also directed for the digit III. However, we keep the name present in the NAV (2017) to avoid problems in the comparative anatomy with other species such as domestic carnivores where it is only directed for these digits, which is constant in *T. mexicana* (our study) and in *T. tetradactyla* (Windle and Parsons, 1899). In *C. didactylus,* it only extends the digits II and III, but only because the digiti I is absent (Galton, 1869a; Humphry, 1869; Macalister, 1875a,b), while in *M. tridactyla* it sends tendons for the digits I and II, and joins to the tendon of the m. extensor digitorum communis (Macalister, 1875a,b). In sloths and armadillos, it does not contribute to the extension of the digit III, but to the other middle digits in a varied way (Macalister, 1869; Galton, 1869a,b; Mackintosh, 1870, 1875; Macalister, 1875a,b; Windle and Parsons, 1899), except in *D. novemcinctus* where was directed for the digit III (Olson et al., 2015).

In *T. mexicana*, the m. extensor digitorum lateralis sent a tendon for the proximal phalanx of the digit I, therefore it should contribute to the function proposed by Machado-Cruvinel et al. (2019), who suggested that the presence of the digit V in *T. tetradactyla* may be related to the ambulation and balance of the hand to support the weight in the lateral side of the hand. We also corroborated the presence of the metacarpal V and its proximal phalanx through gross dissection and radiographs in *T. mexicana*.

The markedly larger development of the craniolateral forearm muscles permits compensation of the flexion strength acquired by the m. flexor digitorum profundus, which has been supported and increased with the fusion of the medial head of the m. triceps brachii in *Tamandua* sp. (Taylor, 1978).

The superficial brachial, transverse cubital and interosseous arteries in *T. mexicana* have been adapted to supply the craniolateral muscles of the forearm with shape of a plexus, which corroborate the major functionality of these muscles in comparison with other species. Even the superficial brachial and cranial superficial antebrachial arteries do not supply these muscles in domestic carnivores and the latter artery is found in the cranial part of the forearm (Ghoshal, 1982; Barone, 2000; Bezuidenhout, 2013), while in *T. mexicana* it was found in the medial part of the forearm. This was similar to the radial artery of primates, since it has a similar trajectory, even sending deep branches between the brachioradialis and brachialis muscles, which are named as radial recurrent artery (Aversi-Ferreira, 2009; Standring, 2016). However, the radial artery in *T. mexicana* was formed from the median artery, similar to domestic carnivores, but in *T. mexicana* the radial artery anastomosed with the medial branch of the cranial superficial antebrachial artery. Wislocki and Straus (1932) reported that the formation of vascular bundles (rete mirabile) from the main vessels of the forearm in *Tamandua tetradactyla* could be due to its largely arboreal life style, and its muscular activity permits movements that are to some extent deliberate and slow, similar to *T. mexicana* but are faster than those of sloths and cyclopes, where the arterial plexuses are more developed.

The formation of rete mirabile in the mammals is a blood cooling system (Scholander, 1955; Castellini, 2009), which permits us to have two hypotheses regarding the development of rete mirabile for the craniolateral forearm muscles in *T. mexicana*: 1) It permits a more efficient blood supply for the muscular activity, and 2) It reduces the temperature produced in calorigenesis by the muscular contraction.

## 5 Conclusions

The northern tamandua (*Tamandua mexicana*) in the craniolateral part of the forearm presents adapted muscles to perform functions such as elbow flexion, supination, carpal and digital extension. The supination has been reinforced by the presence of the m. brachioradialis accessorium, which also contributes to the elbow flexion and should help keep the hand in supination while the animal is supported in a quadrupedal manner. Otherwise, the m. extensor digitorum communis has been developed to sends one tendon only to the digit III, so the strength is concentrated on the most developed digit. There are even muscles that contribute to its function by fusing with its extensor sheath, and these also extend the other digits-however the higher strength is found directed to the supination and extension of the carpus, therefore also to the flexion of the elbow. The anatomical disposition of these muscles varies widely with domestic carnivores, even their functions are support by a major arterial supply, and therefore, it is important to consider these differences for clinical procedures and surgical accesses that must performed from the middle of the arm to the craniolateral part of the forearm and dorsum of the hand.

## Declarations

### Author contribution statement

Paula Valentina Polania-Guzmán: Performed the experiments; Analyzed and interpreted the data; Wrote the paper.

Juan Fernando Vélez-García: Conceived and designed the experiments; Performed the experiments; Analyzed and interpreted the data; Wrote the paper.

### Funding statement

This work was supported by Universidad del Tolima, Colombia.

### Competing interest statement

The authors declare no conflict of interest.

### Additional information

No additional information is available for this paper.

## Figures and Tables

**Fig suppl 1:**
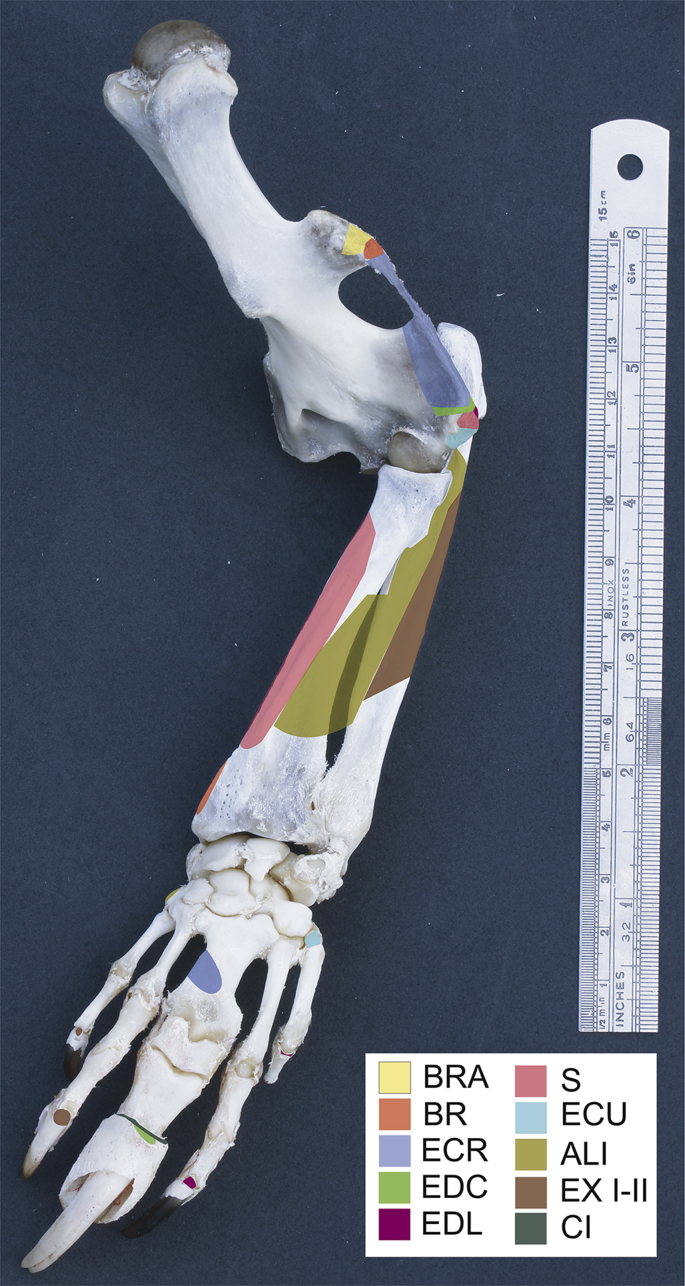


**Fig suppl 2:**
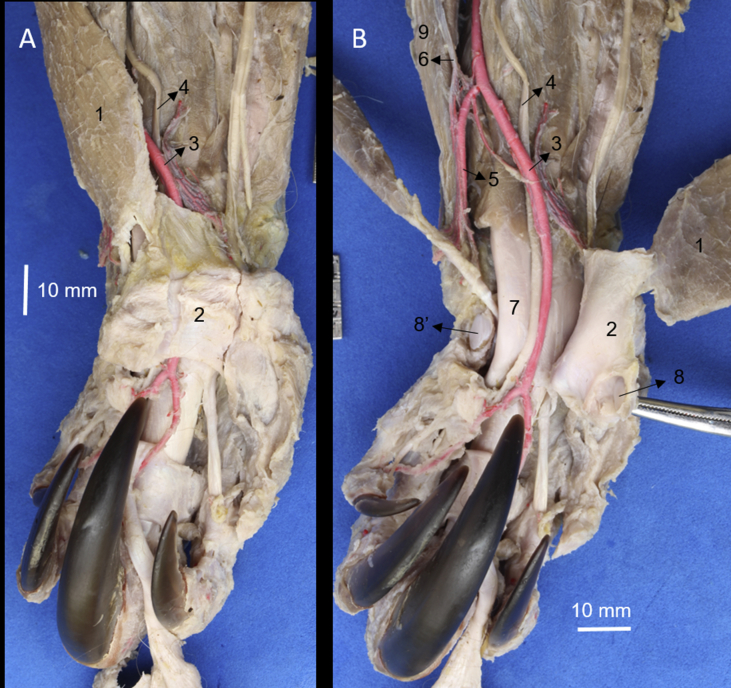


**Fig suppl 3:**
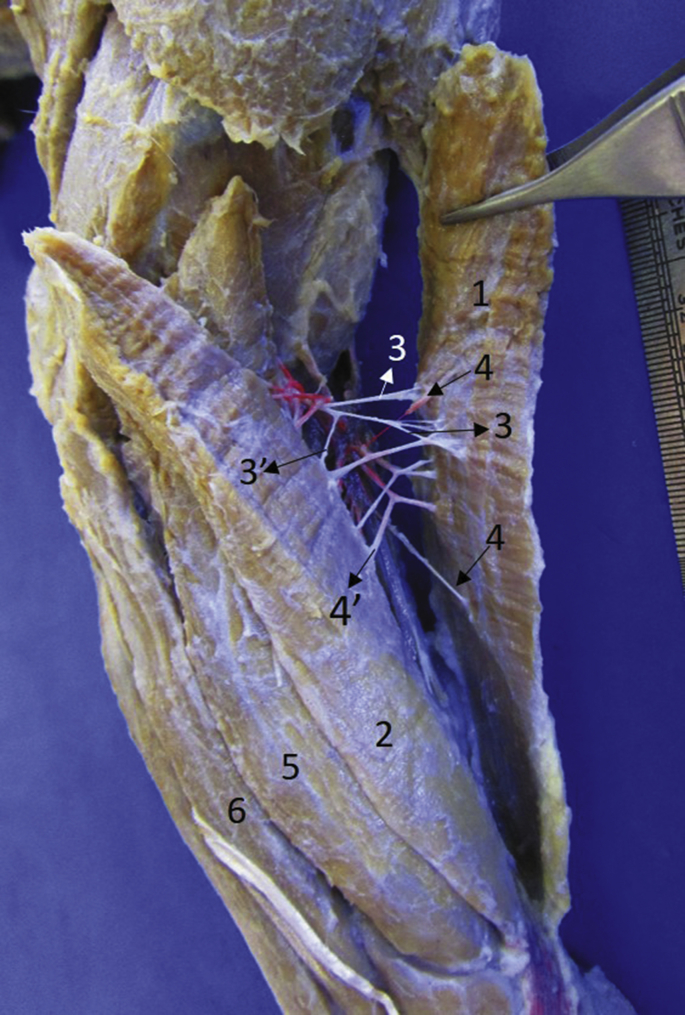


**Fig suppl 4:**